# A Mass Spectrometry-Based Approach for Mapping Protein Subcellular Localization Reveals the Spatial Proteome of Mouse Primary Neurons

**DOI:** 10.1016/j.celrep.2017.08.063

**Published:** 2017-09-12

**Authors:** Daniel N. Itzhak, Colin Davies, Stefka Tyanova, Archana Mishra, James Williamson, Robin Antrobus, Jürgen Cox, Michael P. Weekes, Georg H.H. Borner

**Affiliations:** 1 Department of Proteomics and Signal Transduction, Max Planck Institute of Biochemistry, 82152 Martinsried, Germany; 2 Cambridge Institute for Medical Research, University of Cambridge, Hills Road, Cambridge CB2 0XY, UK; 3 Department of Molecules-Signaling-Development, Max Planck Institute of Neurobiology, 82152 Martinsried, Germany

**Keywords:** spatial proteomics, organellar proteomics, neurons, primary cells, quantitative mass spectrometry, label-free quantification, tandem mass tagging, TMT, LFQ, EGF signaling

## Abstract

We previously developed a mass spectrometry-based method, dynamic organellar maps, for the determination of protein subcellular localization and identification of translocation events in comparative experiments. The use of metabolic labeling for quantification (stable isotope labeling by amino acids in cell culture [SILAC]) renders the method best suited to cells grown in culture. Here, we have adapted the workflow to both label-free quantification (LFQ) and chemical labeling/multiplexing strategies (tandem mass tagging [TMT]). Both methods are highly effective for the generation of organellar maps and capture of protein translocations. Furthermore, application of label-free organellar mapping to acutely isolated mouse primary neurons provided subcellular localization and copy-number information for over 8,000 proteins, allowing a detailed analysis of organellar organization. Our study extends the scope of dynamic organellar maps to any cell type or tissue and also to high-throughput screening.

## Highlights

• High-resolution organellar maps with label-free quantification (LFQ)
• High-throughput organellar maps with TMT-based multiplexing
• Deep mapping of EGF-induced protein localization changes with SILAC, LFQ, and TMT
• A quantitative spatial proteome from mouse primary neurons

## Graphical Abstract

**Figure:**
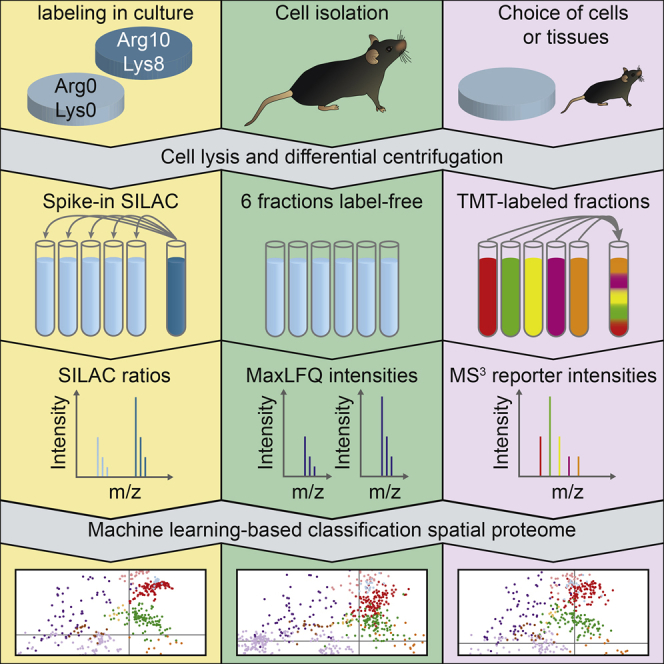

## Teaser

Dynamic organellar maps previously provided a proteomic method for capturing protein subcellular localization changes in cultured cells. Itzhak et al. have now adapted the approach to a universal format, extending the method to all cell types. Application to primary mouse neurons provides spatial and quantitative information for more than 8,000 proteins.

## Introduction

Spatial proteomics is an emerging field that promises to chart the location of all proteins within cells, allowing a systems view of cellular organization (Boisvert et al., 2012, Christoforou et al., 2016, Foster et al., 2006, Hesketh et al., 2017, Itzhak et al., 2016, Jadot et al., 2017, Jean Beltran et al., 2016, Mardakheh et al., 2016, Rhee et al., 2013, Weekes et al., 2014; reviewed in Aebersold and Mann, 2016, Drissi et al., 2013, Jean Beltran et al., 2017, Larance and Lamond, 2015). We have previously developed a profiling method for the generation of highly reproducible organellar maps (Itzhak et al., 2016) that also allows dynamic mapping of induced changes in protein localization. The method combines rapid subcellular fractionation with quantitative mass spectrometry (MS). Because it relies on metabolic labeling (stable isotope labeling by amino acids in cell culture [SILAC]; Ong et al., 2002) for profile quantification, it is mostly suited to cells in culture. To expand the range of applications, here we have developed workflows for label-free quantification using MaxLFQ (Cox et al., 2014) and tandem mass tag (TMT)-based quantification using the MS3/multi-notch approach (McAlister et al., 2012, McAlister et al., 2014). We provide a comparison of the advantages of each method for generating dynamic organellar maps and apply the label-free workflow to neurons, deriving a high-resolution quantitative spatial proteome from primary cells.

## Results and Discussion

### Adaptation of the Dynamic Organellar Maps Workflow

**Figure 1:**
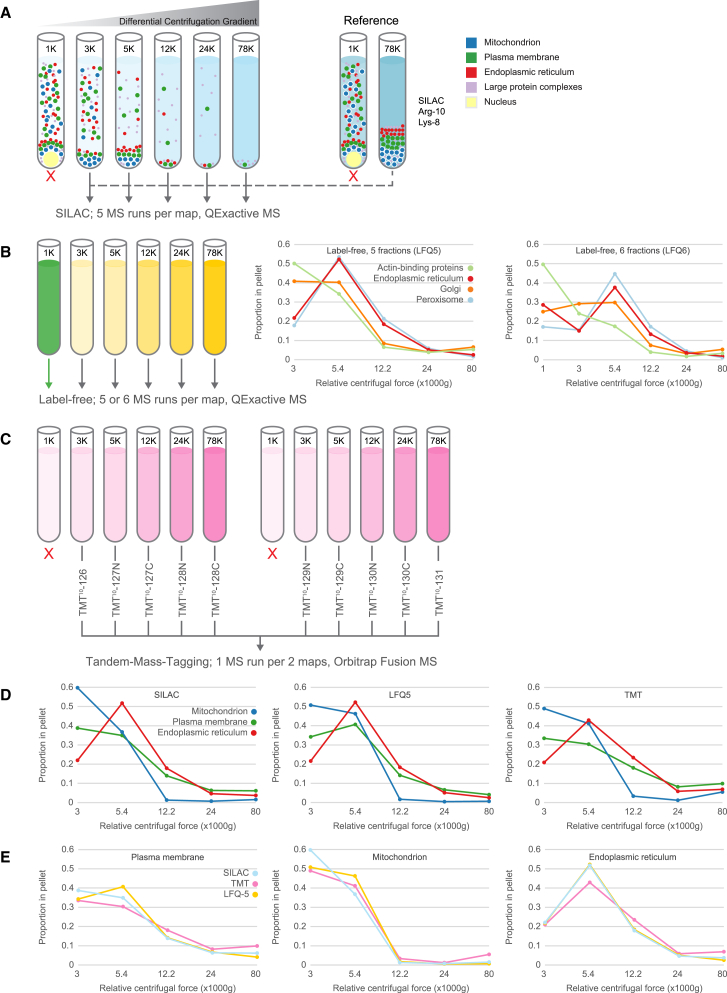
Workflow for Dynamic Organellar Maps Using Fractionation Profiling (A) In all workflows, whole-cell lysate was subjected to differential centrifugation to generate fractions enriched in different organelles. Note that the nuclear-enriched 1K fraction also contains a proportion of non-nuclear material. For the SILAC workflow, heavy-labeled post-nuclear supernatant was subjected to a single centrifugation step to generate a reference membrane fraction. Each of the fractions, excluding the 1K nuclear fraction, was combined 1:1 with the reference fraction and measured by MS. The SILAC ratios along the gradient generate profiles for each protein. In comparative experiments, the SILAC heavy reference fraction was from cells treated to match the fractionated material. (B) LFQ workflow. The same differential centrifugation as for SILAC light was used. Including the 1K nuclear-enriched fraction in the analysis increased separation of some organelles, as seen by comparing median organellar marker profiles (5 fractions, center, versus 6 fractions, right). Please note that inclusion of the 6^th^ fraction also entails re-normalization of the profile to a sum of 1; this causes relative shifts in all fractions. (C) TMT workflow, which used identical fractions as the SILAC light workflow. Following protein digestion, peptides from each fraction were labeled with tandem mass tagging reagent and analyzed on an instrument capable of synchronous precursor selection-MS^3^ (SPS-MS3). TMT 10-plex permitted two maps to be measured in a single experiment. (D) Median profiles for organellar marker proteins are shown for three organelles with the different methods: SILAC (left), LFQ (center), and TMT (right). (E) As for (D), except profiles for the same organelle obtained with the different quantification strategies are shown.

The principle of subcellular proteomic profiling is to partially separate organelles by biochemical means and then to quantify the distributions of proteins across the differentially enriched subfractions. Organelle-specific profiles are derived from the distributions of known marker proteins, enabling subcellular assignment of proteins without known location. Importantly, complete isolation of individual organelles is not required; overlapping profiles can be de-convoluted and resolved by subsequent cluster analysis, provided they are sufficiently different. In the original dynamic organellar maps workflow, cell lysate is separated by differential centrifugation into six fractions (Itzhak et al., 2016). Each of the five post-nuclear pellets is mixed 1:1 with a SILAC heavy “reference” membrane fraction, followed by MS analysis (Figure 1A). Quantification of heavy to light ratios in each fraction yields abundance profiles across the gradient. For label-free quantification (LFQ) implementation, the SILAC workflow was replicated, omitting the heavy-labeled reference (Figure 1B, left). Profiling was then achieved by direct comparison of protein intensities across fractions using the MaxLFQ algorithm for quantification (Cox et al., 2014). With a five-fraction workflow (LFQ5), some organelles showed overlapping profiles. Inclusion of the sixth (nuclear-enriched) fraction (LFQ6) and re-normalization substantially enhanced the resolution of these profiles (Figure 1B, center and right). For a chemical labeling profiling approach, following fractionation and protein digestion, peptides were conjugated with TMT reagent (McAlister et al., 2012, McAlister et al., 2014). Each tag has the same mass but, upon fragmentation, gives rise to reporter ions with different masses; these are used to quantify the abundance of the parent peptides across samples. For maximum accuracy, reporter ions were analyzed with a synchronous precursor selection MS3 approach to avoid ratio compression effects (McAlister et al., 2014).The recent development of 10-plex TMT enabled combination of two maps of five fractions in a single MS run (Figure 1C). With all three profiling strategies, median profiles of major organelles were clearly resolved (Figure 1D). Furthermore, comparing profiles of the same organelle across methods revealed that they were closely matched (Figure 1E).

### Evaluation of SILAC, LFQ, and TMT Map Performance

Map performance for the different quantification strategies was assessed with two MS protocols, a “fast” method that minimizes measuring time and a “deep” method that maximizes protein coverage. These reflect run parameters we anticipate will be employed by users. The MS measurement requirements for SILAC and LFQ5 were identical (12.5 hr/fast map, 37.5 hr/deep map), and substantially lower for TMT (1.5 hr/fast map, 19 hr/deep map) because of the multiplexing of samples.

**Figure 2:**
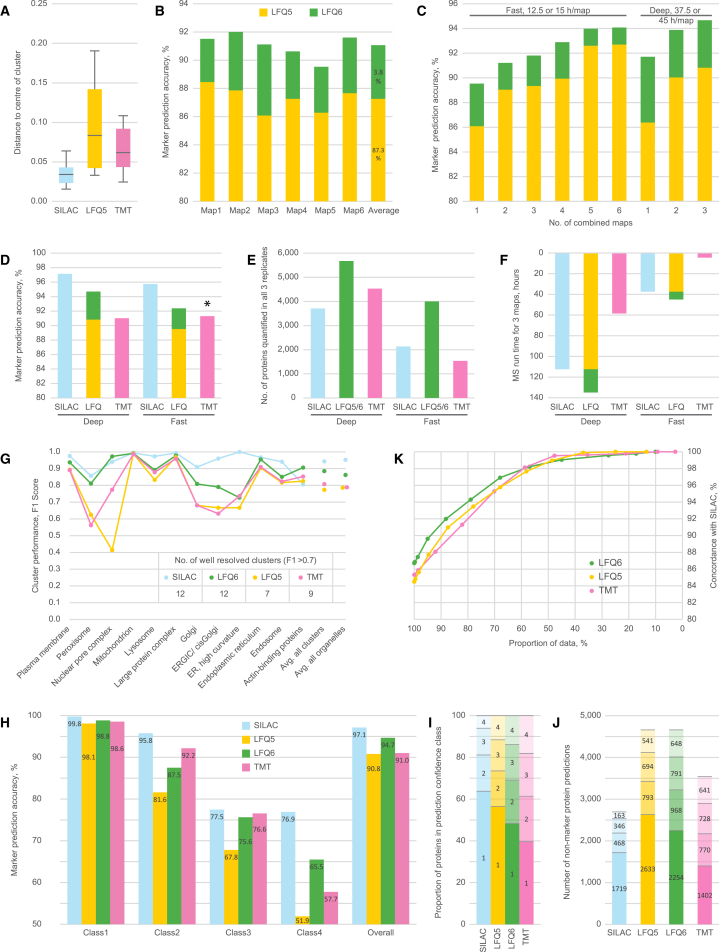
Performance Analysis of Organellar Maps Generated with TMT, LFQ, and SILAC Quantification Strategies (A) To illustrate the relative precision of the different quantification methods applied in fractionation profiling, profile scatter within the 20S core proteasome (14 subunits, PSMA1–7, PSMB1–7, three independent measurements per protein) was analyzed (deep MS protocol). LFQ measurements are “noisier” than SILAC or TMT. Boxes indicate the interquartile range and whiskers 10^th^–90^th^ percentile range. (B) Organellar classification performance of six independent LFQ-based maps. Accuracy is the proportion of correctly classified organellar markers during supervised learning. Performance was assessed for six-fraction profiles (LFQ6, green) and for the same maps with the sixth data point removed (LFQ5, yellow). (C) Combining several LFQ maps for organellar classification enhanced prediction accuracy. (Fast) maps shown in (B) were combined in the order of lowest to highest performance. Addition of each map improved performance. Maps 3, 4, and 6 were then chosen for further deep MS analysis and combined for classification. (D) Marker prediction accuracy obtained with a combination of three replicate maps by quantification strategy and MS protocol. TMT fast maps included predictions for only 10 of 12 clusters (see also Figure S1G). (E) Number of profiled proteins quantified in all three replicates. (F) MS measurement requirements (hours) for the generation of three replicate maps. (G–K) In-depth analysis of the predictions obtained with a combination of three replicate datasets, deep MS protocol (an equivalent analysis for predictions obtained with the fast MS protocol is shown in Figures S1B–S1E). (G) Detailed performance profiles of maps made with SILAC, LFQ5/6, and TMT. Prediction performance was evaluated for each organellar cluster. F1 scores were calculated as the harmonic mean of recall (true positives / [true positives + false negatives]) and precision (true positives / (true positives + false positives]). High F1 scores (> 0.7) denote clusters with a high predictive value. (H) Stratification of non-marker organellar predictions. Each assignment was made with a prediction confidence score. Four different SVM score cutoffs were defined, dividing the data into confidence classes. The prediction accuracy of marker proteins within each class served as a proxy for the prediction accuracy of non-marker proteins. Generally, the first two classes had high accuracies with all methods. (I and J) Proportion (I) and absolute number (J) of non-marker predictions in each confidence class. (K) Concordance analysis. The predictions of non-marker proteins, obtained with TMT, LFQ5, and LFQ6, were compared with the predictions obtained with SILAC. Concordance is the proportion of proteins with identical predictions. Restricting the comparison to proteins with a minimum confidence score in both compared maps reduces the overlapping dataset but increases concordance. In all cases, over 85% of the predictions show > 90% agreement. See also Figure S1 and Table S1.

It was expected that the LFQ implementation would be most challenging because of the noisier quantification relative to SILAC or TMT (Figure 2A); hence, the LFQ approach was optimized most extensively. Six independent LFQ maps were prepared from HeLa cells with the fast MS protocol. Data transformation and quality filtering were adjusted for LFQ profiles as detailed in the Supplemental Experimental Procedures. Organellar predictions were generated using supervised learning (support vector machines [SVMs]) of a set of approximately 1,000 marker proteins covering 12 subcellular localizations (Itzhak et al., 2016). The proportion of accurately assigned markers was scored (global prediction accuracy; Figure 2B). The average map performance for LFQ5 (fast) was 87.3%. Inclusion of the sixth fraction led to a consistent and substantial boost in prediction accuracy, taking performance to an average of 91.1% for LFQ6. For reference, SILAC (fast) maps average ∼94% accuracy.

Organellar classification using the combined profiles of several SILAC maps enhances performance (Itzhak et al., 2016). To investigate this effect with LFQ, classification was performed with one to six LFQ (fast) maps, combining them in order of performance from worst to best (Figure 2C). Each additional map improved the performance, plateauing at 5 maps (prediction accuracy, ∼94% for LFQ6). Three maps of intermediate performance were selected for more extensive MS analysis (deep protocol). This revealed that two deep LFQ maps combined had equivalent prediction accuracy as five fast maps (Figure 2C). An equivalent analysis was performed for TMT maps (single maps versus a combinations of maps, fast versus deep protocols) as well as for SILAC (to serve as a reference; Figure S1). In all cases, a combination of three maps provided high-accuracy organellar predictions (Figure 2D). Using the deep protocol, SILAC provided the best global prediction accuracy at 97.1%; LFQ5 and TMT maps had slightly lower accuracies (around 91%) but were still very good in absolute terms. The boost from including the extra fraction placed LFQ6 performance close to SILAC (94.7%). The number of profiled proteins was lowest with SILAC (3,700), whereas that with LFQ exceeded 5,500 (Figure 2E). With TMT, 4,500 proteins were profiled; however, two of three replicates covered more than 6,000 proteins, suggesting that the depth should reach that of LFQ maps. The fast protocol provided a slightly lower map accuracy in all cases, but it was still very high for SILAC (95.8%) and LFQ6 (92.4%). TMT fast also had good accuracy (91.3%), although this was calculated for a smaller set of resolved clusters (Figure 2D; Figure S1G). MS measuring time requirements were substantially lower with TMT quantification, especially with the fast protocol (only 4.5 hr/three maps; Figure 2F).

For in-depth performance analysis of maps generated with the different quantification methods, the predictions for individual organellar clusters were evaluated. We calculated recall (the proportion of marker proteins correctly assigned to the cluster) and precision (the proportion of all assignments to this cluster that are correct). A perfectly resolved cluster includes all relevant marker proteins and no markers from any other clusters (recall and precision = 1). The harmonic mean of recall and precision, the F1 score, provides a single metric of cluster performance. A comparison of the different methods revealed that some clusters perform well irrespective of the MS acquisition method (Figure 2G); these included the largest clusters: plasma membrane, mitochondrion, endoplasmic reticulum, and large protein complex as well as endosome, lysosome, and actin-binding proteins. Smaller clusters, including peroxisome, nuclear pore complex, Golgi, and ER-Golgi intermediate compartment (ERGIC), performed less well in TMT and LFQ5 compared with SILAC. The benefit of LFQ6 relative to LFQ5 was also most evident for these clusters. Defining an F1 score of > 0.7 as a well-resolved cluster, both SILAC and LFQ6 resolved all 12 clusters, suggesting that these are the preferred methods for the highest-resolution maps; although not directly tested here, a TMT-based deep analysis with 6 fractions would be likely to yield results similar to LFQ6 (Figure 2G). Figures S1F–S1I show how the F1 scores improve when using the deep protocol compared with the fast protocol.

Organellar predictions of non-marker proteins were stratified into four confidence classes based on SVM scores (high, medium, low, and very low). Marker prediction accuracies within each class served as a proxy for the prediction accuracy of non-markers (Figure 2H). SILAC had the greatest proportion of high-confidence predictions, but TMT and LFQ also had high proportions (Figure 2I). Overall, LFQ made the largest number of high-confidence predictions because of the overall number of proteins profiled (Figure 2J; Figures S1C–S1E show the equivalent analyses for maps made with the fast protocol).

Finally, it was evaluated to what extent the organellar assignments made with the different methods agree. Concordance was calculated as the proportion of proteins with identical predictions between two quantification methods. For each comparison, the SILAC (deep) set was used as reference. Importantly, only non-maker predictions were included in the analysis. Baseline concordance was very high in all cases (84%–87%; Figure 2K; Figure S1B). A stringency filter was then applied to restrict comparisons to predictions above a given SVM score. In all cases, concordance reached >96% for the majority of predictions, demonstrating that the three profiling methods yield highly consistent results. Thus, we conclude that the SILAC, LFQ, and TMT quantification strategies are all effective for generating accurate organellar maps.

### TMT- and LFQ-Based Dynamic Organellar Maps

We next investigated the suitability of TMT and LFQ maps to capture induced protein translocations. For optimum comparison, an identical set of samples, comprising three replicate experiments of control cells or cells stimulated with epidermal growth factor (EGF) for 20 min, was analyzed with all three methods using both fast and deep protocols. These samples were used previously to follow endocytic uptake of activated EGF receptor (EGFR) but were analyzed only with the fast SILAC protocol (Itzhak et al., 2016). Here, an additional deep MS analysis was performed to determine the full capability of the SILAC approach. To test LFQ maps for dynamic applications, a label-free experiment was simulated by reprocessing the SILAC fast and deep datasets with the MaxLFQ algorithm, ignoring any SILAC heavy-labeled peptides. For TMT dynamic maps, peptides from the SILAC light fractions were TMT-labeled and analyzed by MS (fast and deep protocols).

**Figure 3:**
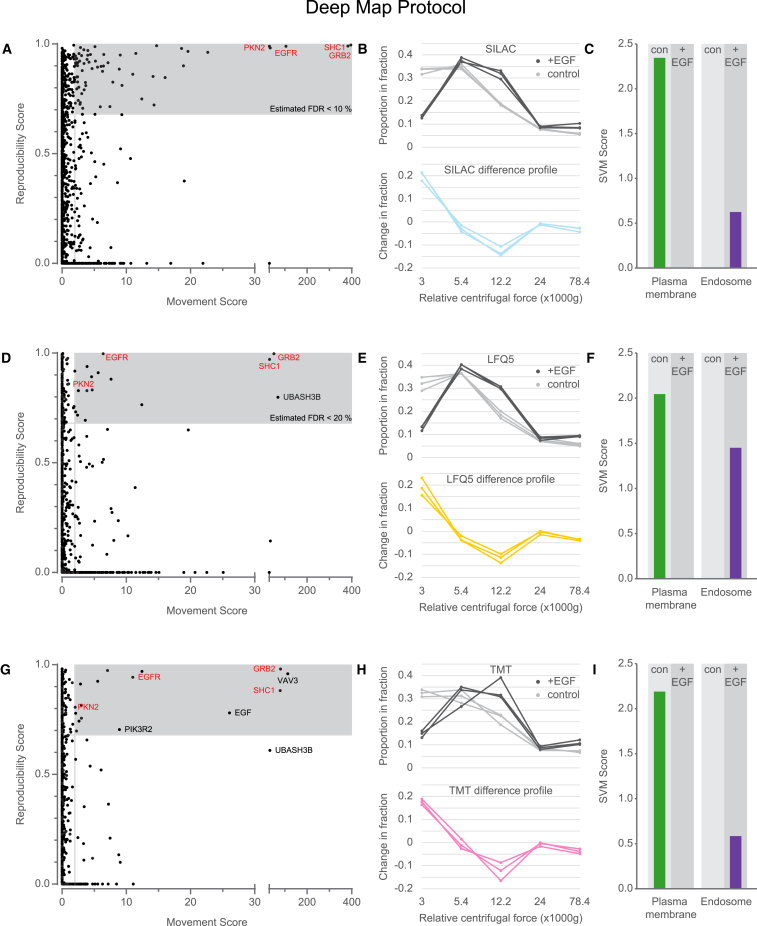
Assessment of Dynamic Organellar Maps with Different Quantification Strategies Using the Deep MS Protocol (A) Three replicate SILAC experiments of cells left untreated or stimulated with EGF for 20 min were analyzed in a dynamic organellar maps experiment. The resulting difference profiles were subjected to statistical analysis to identify moving proteins (see Experimental Procedures for details). The movement and reproducibility scores for each protein are shown in an MR scatterplot; significantly moving proteins have high scores in both dimensions. The shaded area contains proteins where the estimated false discovery rate (FDR) for translocation is < 10% based on a mock control experiment. (B) Top: the proportion of EGFR in each fraction across the differential centrifugation gradient for three replicates in control cells (gray lines) or cells stimulated with EGF (black lines). Bottom: the difference in protein pelleting in the fractions in untreated compared with EGF-treated cells for three replicates. (C) Proteins in the shaded area of (A) were removed from the marker set, and all remaining proteins were subjected to organelle classification using SVM-based machine learning. The prediction scores for the plasma membrane and endosome are shown before and after treatment with EGF, correctly capturing the change in localization of the EGF receptor. (D–F) The same as (A)–(C), respectively, but for LFQ-based (deep) experiments. Note that the shaded area corresponds to a translocation FDR of < 20%. (G–I) Also the same as (A)–(C), respectively, but using data from the TMT-based (deep) experiments. Note that the shaded area is not FDR-controlled but uses cutoffs determined for the SILAC and LFQ experiments. See also Figures S2 and S3 and Table S2.

To identify proteins that show subcellular movement upon EGF treatment, an improved version of our previously developed outlier test was applied (Supplemental Experimental Procedures). This combines metrics for movement distance (M score) and reproducibility (R score) into an “MR” scatterplot analysis. Significantly translocating proteins have both high M and R scores. False discovery rate (FDR) control for cutoff selection was achieved by comparison with a mock experiment (control versus control). These plots revealed that SILAC, TMT, and LFQ implementations of dynamic organellar maps correctly identified the movement of EGFR together with SHC1 and GRB2, two major binding partners of activated EGFR (Figures 3A, 3D, and 3G). The profiles of the EGFR, before and after treatment with EGF (Figures 3B, 3E, and 3H), were remarkably similar across all methods. Furthermore, when subjecting each of the datasets to SVM analysis, all methods correctly classified EGFR as localized to the plasma membrane in control cells and to endosomes in EGF-treated cells (Figures 3C, 3F, and 3I). Importantly, almost identical results were obtained with the corresponding fast analyses (Figure S2), also highlighting the usefulness of all methods in this format.

Although all three approaches successfully identified major translocations, they differed in the number of detected minor movements (Figure S3). Here, SILAC performed best, identifying a total of 66 significant translocations (with an estimated FDR < 10%). 42 of these have previously been linked to EGF signaling, strongly supporting the high predictive value of the analysis; the remaining proteins are hence likely candidate pathway components or downstream targets of EGFR (see Figure S3 and Table S2 for complete annotation). TMT and LFQ maps both detected sixteen movements but, in the case of LFQ, with a higher FDR. Of note, the improved depth of LFQ maps enabled the identification of UBASH3B movement, a protein absent from the SILAC dataset. Conversely, TMT was the only method to identify movement of EGF; this protein was not present in control cells and, hence, was excluded from LFQ and SILAC analyses, but, because of multiplexing of two maps, the TMT approach can handle such cases.

**Figure 4:**
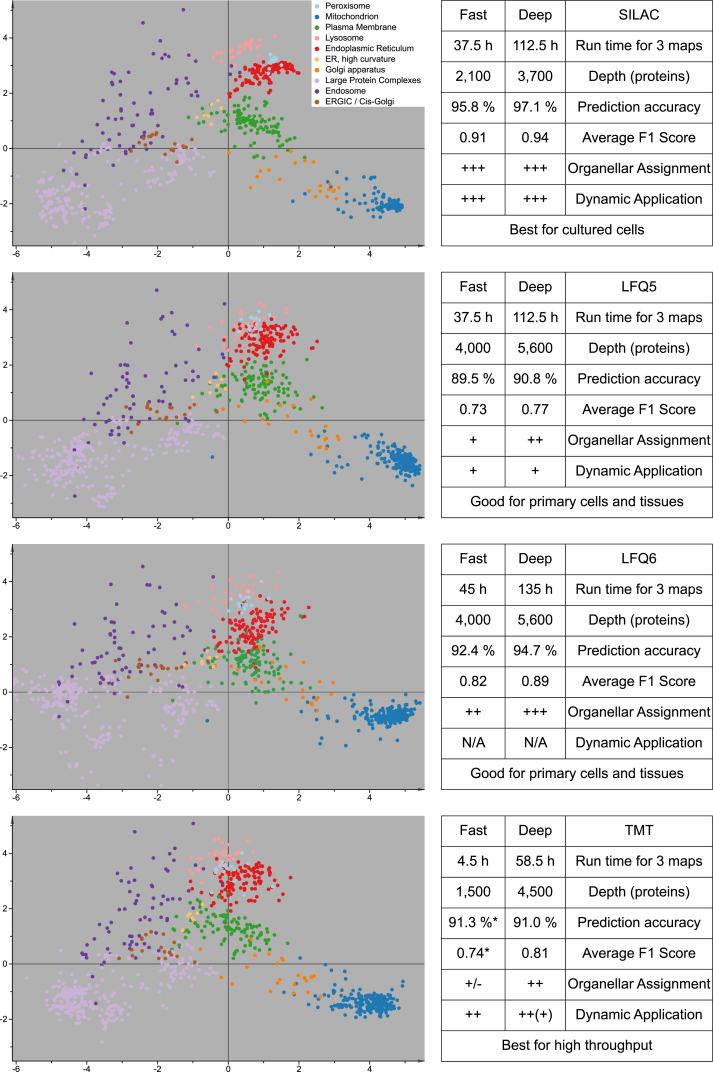
Visual Map Representation of 941 Marker Proteins Common to All Triplicate Deep Datasets (Left) and Key Metrics and Characteristics for Both Fast and Deep MS Protocols of the SILAC, LFQ5, LFQ6, and TMT Methods (Right) Plots for the SILAC, LFQ5, and TMT methods were generated from a single principal-component analysis, where each marker protein had three different entries, one for each of the methods, and each entry had fifteen data points corresponding to three replicates of five fractions. Because LFQ6 has an additional data point for each map, an independent PCA was used to generate this plot; it was then scaled for optimum comparison with the other methods. All maps show highly similar separation and orientation of marker protein clusters, with increased cluster density of SILAC relative to other methods, most evident with the peroxisomal cluster. Furthermore, note that each plot is a 2D representation of a 15-dimensional dataset (18-dimensional for LFQ6); many seemingly overlapping clusters are resolved in higher dimensions not illustrated here. TMT fast maps include predictions for only 10 subcellular localizations; all other maps include 12.

Key metrics and characteristics for static and dynamic applications of each method are summarized in Figure 4.

### Application of LFQ Organellar Maps to Mouse Neurons

**Figure 5:**
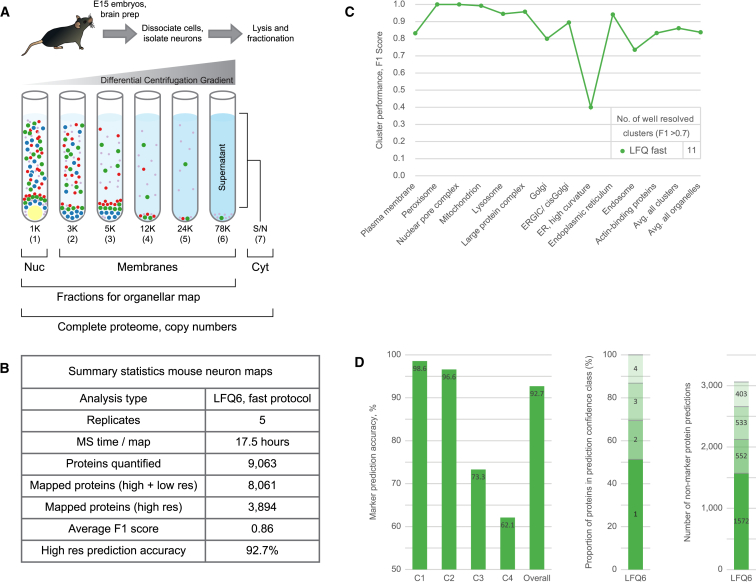
Application of Label-free Organellar Mapping to Mouse Neurons (A) Schematic workflow. Cortical neurons were acutely isolated from embryonic mice, lysed mechanically, and subjected to a series of differential centrifugation steps: 1, nuclear-enriched fraction; 2–6, membrane fractions; 7, cytosol. All fractions were analyzed by label-free quantitative mass spectrometry. Fractions 1–6 were used to generate organellar maps. Fractions 1, 2–6 combined, and 7 were used to quantify proteins’ nuclear, membrane-associated, and cytosolic pools. All fractions, 1–7 combined, were used to calculate protein copy numbers per cell. (B) Summary of neuron map performance (combined output from five independent replicates). (C) Detailed performance profiles of neuron maps. F1 scores were calculated as the harmonic mean of recall and precision, for each compartment, as in Figure 2G. (D) Stratification of non-marker organellar predictions as in Figure 2H. The prediction accuracy of marker proteins within each class served as a proxy for the prediction accuracy of non-marker proteins. The first two classes had very high accuracies. Proportion and absolute number of non-marker predictions in each confidence class are shown in the center and on the right, respectively. See also Figure S4 and Tables S3, S4, and S5.

The successful implementation of LFQ organellar maps opened the possibility to investigate the spatial proteome of primary cells. To test this, we prepared acutely isolated neurons from embryonic mice (sacrificed at embryonic day 15 [E15]). At this stage of development, neurons show relatively little neurite arborization, which facilitates their isolation (Sciarretta and Minichiello, 2010). In total, five independent replicates were prepared on three separate days. Cells were lysed mechanically and subjected to our standard differential centrifugation scheme (Figure 5A). In addition to the six membrane fractions (LFQ6), we also collected the cytosol; this allowed us to capture the complete spatial and quantitative proteome from a single workflow despite very limited amounts of starting material (only 1–2 mg of protein/preparation). Samples were analyzed with the fast MS protocol (17.5 hr/preparation). In total, over 9,000 proteins were identified (Table S3). The combined output from all five replicates was then jointly processed to generate organellar maps; 3,894 proteins were profiled across all replicates. These were annotated with the same set of organellar markers as for HeLa cells, without any further cell-specific optimization (834 markers matched across species). Application of SVM machine learning showed a high overall marker prediction accuracy of 92.7% (with full cross-validation; Figure 5B). For a more detailed performance evaluation, we calculated F1 scores for each compartment cluster (Figure 5C). 11 of 12 clusters showed high resolution, with the exception of the (rather minor) endoplasmic reticulum (ER)-high curvature cluster. Stratification of the prediction classes (Figure 5D) revealed a large proportion of high-confidence predictions. Collectively, these data show that the performance of the LFQ neuron maps is extremely similar to what we had previously observed in HeLa cells (Figure 2; Figure S1) and demonstrate that the LFQ protocol is suitable for application to primary neurons.

In addition to the organellar localization data, our analysis also provided information on the global distribution across the membrane, nuclear, and cytosolic fractions for over 6,000 proteins. These included 1,120 proteins classified as mostly nuclear, 1,471 as mostly cytosolic, and 528 as nuclear and cytosolic (Table S4). Finally, we derived absolute protein abundances (i.e., copy numbers and cellular concentrations) for over 9,000 proteins using the proteomic ruler approach (Wiśniewski et al., 2014; Figure S4). Together, these data provide a comprehensive account of the mouse cortical neuron spatial proteome (Table S4).

### A Quantitative Comparison of Mouse Neuron and HeLa Organellar Organization

**Figure 6:**
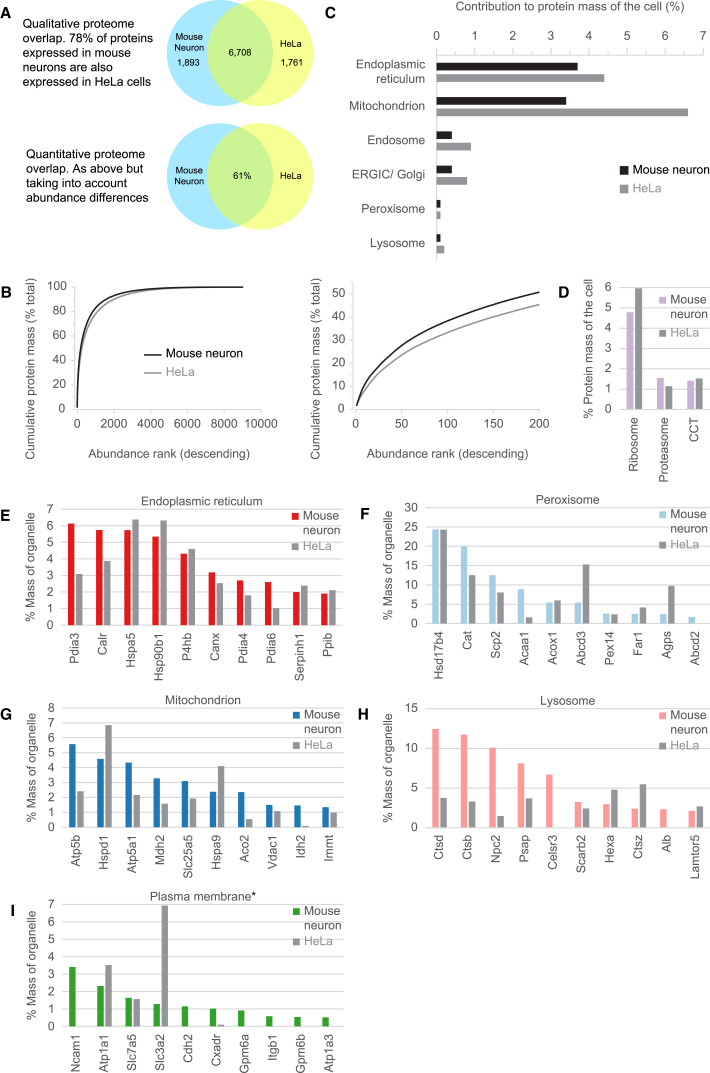
Comparative Organellar Anatomy of Mouse Neurons and HeLa Cells (A) Full proteome overlap analysis. Top: qualitative overlap; the proportion of proteins detected in mouse neurons, with orthologs expressed in HeLa cells. Bottom: quantitative overlap (protein IDs and abundance considered). (B) Proteins detected in neurons (black) or HeLa cells (gray) were ordered by abundance. The cumulative contribution to total cell protein mass was plotted on the y axis. In both cases, the 100 most abundant proteins contribute over one-third of the total protein mass (right). (C) Relative contribution of individual organelles to total cell protein mass. Please note that the mouse neurons were acutely isolated; during this procedure, neurites, and, hence, parts of the plasma membrane, are lost (see Supplemental Experimental Procedures for details). This will lower the apparent plasma membrane contribution (which is not shown here for this reason) but is unlikely to substantially affect other organelles. (D) Abundant protein complexes make remarkably similar contributions to the total proteome in both cell types. CCT, a multi-subunit chaperonin, is also known as TRiC. (E–I) Compositional analysis of major organelles: (E) ER, (F) peroxisome, (G) mitochondrion, (H) lysosome, (I) plasma membrane. In each case, the ten most abundant proteins of the neuronal organelle were determined; the y axis shows their contributions to the total mass of the organelle. For comparison, the contributions of the same proteins to the corresponding HeLa organelles are shown (gray bars). Some organelles have extremely similar compositions (e.g., ER, peroxisome), others differ qualitatively (plasma membrane) or quantitatively (i.e., the same proteins but different distribution; e.g., lysosome). For the plasma membrane, only integral membrane proteins were considered. Although many synaptic marker proteins were detected in neuron lysates (Table S4), we did not observe a separate cluster corresponding to synapses.

The combined knowledge of protein abundance and subcellular localization data allows the reconstruction of cellular anatomy, as we have shown previously for HeLa cells (Itzhak et al., 2016). We prepared an equivalent analysis for primary mouse neurons (Figure 6). We derived a quantitative total proteome (Table S4), the contribution of every organelle to the whole cell protein mass, and also determined the protein composition of individual organelles. The availability of two spatial proteomes, HeLa and mouse neurons, prepared with the same approach and comparable depth of analysis, offered a unique opportunity for a systematic comparison of two very different cell types at the organellar level. HeLa cells are fast-growing immortal cells derived from a cervical carcinoma and are maintained in culture, whereas the neurons were differentiated mouse primary cells freshly isolated from the brain and had never been exposed to culture conditions. We sought to determine to what extent these differences are reflected at the compositional level.

At the qualitative proteome level, 78% (6,700) of all proteins detected in the neurons were also expressed in HeLa cells (assuming that proteins with the same name have orthologous functions in both organisms; Figure 6A). Our proteomic ruler data estimated that HeLa cells were approximately six times larger than the neurons. Factoring in relative protein abundance (copy numbers weighted by protein molecular weight and scaled by cell size), the composition overlap by protein mass drops to around 61%, demonstrating that quantitative and qualitative differences in protein expression both contribute substantially to cellular identity. Conversely, the perhaps surprisingly large degree of overlap suggests that, regardless of cell type, a considerable proportion of the proteome is relatively invariant. Similarly, in both cell types, the 100 most abundant proteins contribute over 30% of the total protein mass (Figure 6B).

We next compared the relative abundance of individual organelles (Figure 6C). In both cell types, mitochondria and the ER were the predominant organelles. For mitochondria, the contribution to total cell protein mass was almost double in HeLa cells (6.6% versus 3.4%), perhaps reflecting their increased need for energy to support continuous growth. In contrast, the ER contributed very similarly in both cells (3.7% in neurons and 4.4% in HeLa cells). The Golgi, endosomes, and lysosomes all made relatively minor overall contributions (all < 1%), although each of these organelles contributed ∼2× greater mass to HeLa cells compared with neurons. The levels of ribosomes (approximately 5%–6%) and proteasomes (approximately 1%–1.5%) were remarkably similar (Figure 6D).

To facilitate the analysis of individual organelles, we identified the ten most abundant proteins in neuron organelles, which, in each case, make up a large proportion of the total organelle mass. We then compared the compositional overlap (by percent protein mass) with the corresponding HeLa cell organelles (Figures 6E–6I). As expected, the plasma membrane composition was radically different, both qualitatively and quantitatively, supporting the notion that the cell surface is a key factor in determining cellular identity (Sharma et al., 2015). Lysosomes also have very different compositions, but the differences are mostly quantitative; the neuronal lysosome is predominated by two cathepsins (Ctsb and Ctsd) that contribute 25% of the proteome, suggesting a specialized role for this compartment. In contrast, the ER has an almost identical composition in both cell types, suggesting that abundant ER constituents are indeed “housekeeping” proteins with similar concentrations across cell types. Of note, peroxisomes are also extremely similar in both cell types and dominated by the same protein, HSD17b4 (beta-hydroxysteroid dehydrogenase), which contributes 25% of the protein mass. Mitochondria show considerable compositional overlap but with specific metabolic adaptations (e.g., complete lack of CPS1 in neurons, a key component of the urea cycle and a major mitochondrial protein in HeLa cells; Itzhak et al., 2016). Although the levels of heat shock proteins are very similar in the ER (both approximately 20%), they are substantially lower in the mitochondria of neurons (approximately 9% versus 14% total); this may again relate to the high biosynthetic load imposed by rapidly growing HeLa cells. Thus, our analysis reveals qualitative and quantitative differences between neuronal and HeLa organelles but also a remarkable set of conserved features.

### Outlook

Here we have established that SILAC, LFQ, and TMT are all highly effective for generating dynamic organellar maps through fractionation profiling, widely extending the scope of this method (summarized in Figure 4; Table S5). LFQ- and TMT-based profiling allow application to primary cells and tissues. As demonstrated for mouse neurons, the LFQ6 format is particularly useful is this regard because of its excellent prediction accuracy. We expect that a sixth fraction would also improve the prediction accuracy for TMT (using, for example, TMT 6-plex) but at the expense of the ability to place two maps in a single TMT 10-plex experiment. Conversely, using the protocols illustrated here, TMT maps required only ∼50% (deep) or 12% (fast) of MS time compared with their SILAC or LFQ equivalents. Multiplexing is the biggest advantage of the TMT approach; with the fast protocol, a triplicate comparative analysis can be performed in as little as 9 hr of total MS measurement time, paving the way for high-throughput spatial proteomics experiments. For cells amenable to metabolic labeling, the SILAC approach offers exceptional performance both for organellar classification and for capture of translocation events. As reported previously (Itzhak et al., 2016) and as shown here, protein copy numbers estimated from the map data can be assigned to organellar proteomes to provide global cellular anatomy; all map formats are equally compatible with this approach.

## Experimental Procedures

Please refer to the Supplemental Experimental Procedures for complete details.

### Analyzed Samples

For this study, we prepared multiple organellar maps from new samples but also re-analyzed several previously generated samples (Itzhak et al., 2016), either with new labeling and MS or new processing (see Supplemental Experimental Procedures for a complete description).

### Cortical Neuron Preparation

Mice (C57BL/6 background) were housed in a specific pathogen-free (SPF) facility with a 12:12 hr light/dark cycle and food and water available ad libitum. All animal experiments were performed in compliance with institutional policies approved by the government of upper Bavaria. For preparation of cortical neurons from embryonic mice (E15), the procedure described in Meberg and Miller (2003) was adapted. This method yields fairly pure neuronal populations (Xu et al., 2012) because glial cells have not developed at this stage (Qian et al., 2000). Furthermore, these neurons have not yet formed extensive dendritic or axonal arbors and can therefore be isolated with relatively little cell damage (Sciarretta and Minichiello, 2010). In total, five independent preparations were analyzed by organellar mapping.

### Subcellular Fractionation Procedure for Label-free Organellar Maps

Cell lysis and subcellular fractionation were performed as reported previously (Itzhak et al., 2016) and as shown in Figure 1 but omitting any steps relating to the SILAC heavy-labeled reference sample. Each map was prepared from a single, ∼70% confluent 15-cm dish of HeLa cells.

### MS

Mass spectrometric analysis of LFQ and SILAC samples was performed with a Q Exactive HF (Thermo Fisher Scientific, Germany), as described previously (Itzhak et al., 2016). For samples in the TMT workflow, MS was performed with an Orbitrap Lumos or an Orbitrap Fusion instrument (Thermo Fisher Scientific, San Jose, CA).

### Processing of MS Data

Raw files were processed with MaxQuant version 1.5 (Cox and Mann, 2008, Tyanova et al., 2016a) using the human or mouse reference protein datasets downloaded from UniProt (SwissProt canonical and isoforms database).

### Statistical Methods

#### Generation of Organellar Maps

Each map experiment generated an abundance distribution profile across the subcellular fractions for every quantified protein; typically, several thousand proteins were profiled in an experiment. To allow cluster analysis, established marker proteins of various subcellular compartments were then identified from a previously defined set (Itzhak et al., 2016). For unsupervised clustering and data visualization, profiles were subjected to principal-component analysis (PCA) (Figure 4). For unbiased and rigorous organellar assignments, the SVM-based supervised learning approach described in Itzhak et al. (2016), implemented in Perseus software (Tyanova et al., 2016b), was then applied. Conceptually, SVMs derive non-linear boundaries between multivariate data clusters. The SVMs were first trained with the marker protein profiles (using cross-validation to prevent overfitting). Non-marker proteins were then assigned to compartments based on the boundaries defined by the markers.

#### Detection of Dynamic Changes between Organellar Maps

The detection of protein translocations followed the procedure established in Itzhak et al. (2016), with several improvements and adaptations for the LFQ and TMT workflows (refer to the Supplemental Experimental Procedures for complete details). Briefly, the analysis is based on a two-tiered statistical test and fully FDR-controlled. First, for each protein, the two five-point profiles obtained from a pair of control and EGF treatment maps are subtracted to obtain a delta profile. All delta profiles are collected in a matrix, and for each delta profile, the robust Mahalanobis distance to the matrix center is calculated. The Mahalanobis distance approximately follows a chi-square distribution with five degrees of freedom and can therefore be converted into a p value (the likelihood to observe a profile as far or farther from the center). In total, three replicate pairs of control and EGF treatments were analyzed. For each protein, three p values for profile shifts were thus obtained. For a stringent analysis, the highest p value from the three replicates was chosen (corresponding to the smallest observed shift). This value was then cubed (because there were three independent replicates, each with a p value smaller or equal to the chosen one) and corrected for multiple hypothesis testing using the Benjamini-Hochberg method. The negative log10 of the corrected p value was the protein’s M score (“magnitude” of movement). Large M scores correspond to large profile shifts. Second, the reproducibility of profile shifts was assessed. For each protein, the Pearson correlation between the delta profiles of replicates 1 versus 2, 1 versus 3, and 2 versus 3 was calculated. Of the three obtained R values, the lowest one was chosen and represents the R score (“reproducibility” of movement). Large R scores correspond to reproducible profile shifts. Genuinely translocating proteins have high M and R scores.

To achieve FDR control, data from a previous “mock” experiment (Itzhak et al., 2016) were used. Six control maps were split into three pairs and analyzed as described above. No genuine translocations were expected here. Applying the same M and R score cutoffs to the EGF treatment data and the mock data yielded the FDR, as the number of hits observed in the mock experiments divided by the number of hits in the EGF treatment experiments (scaled by the relative sizes of the datasets).

#### Software for Statistical Analysis and Graphics

Statistical analyses, data transformation, and filtering were performed in Perseus (Tyanova et al., 2016b), Prism 6 (GraphPad), and Microsoft Excel. Principal component analysis was performed in SIMCA 14 (Umetrics/MKS).

#### Copy-Number Determination and Organellar Composition Analysis

Copy numbers per cell, protein concentrations, and cell volumes were estimated with the proteomic ruler approach (Wiśniewski et al., 2014), implemented in Perseus software (Tyanova et al., 2016b). Organelle composition analysis was performed essentially as described in Itzhak et al. (2016).

#### Webpage

We have improved the web interface for our database of human subcellular localization predictions (http://www.MapOfTheCell.org).

## Author Contributions

Conceptualization, D.N.I., C.D., M.P.W., and G.H.H.B.; Methodology, D.N.I., C.D., A.M., J.C., M.P.W., and G.H.H.B.; Formal Analysis, D.N.I. and G.H.H.B.; Investigation, D.N.I., C.D., R.A., and J.W.; Writing, D.N.I. and G.H.H.B.; Visualization, D.N.I. and G.H.H.B.; Website, S.T.; Supervision, M.P.W. and G.H.H.B.

## Supplemental Information

Document S1. Supplemental Experimental Procedures, Figures S1–S6, and Tables S2 and S5

Table S1. Proteomic HeLa Data Generated in This Study, Related to Figure 2

Table S3. Proteomic Mouse Neuron Data Generated in This Study, Related to Figure 5

Table S4. The Mouse Spatial Proteome (Protein Subcellular Localization Predictions, Copy Numbers, and Cellular Concentrations for More Than 9,000 Proteins), Related to Figure 5

Document S2. Article plus Supplemental Information
