# Supplementary material for: A Mass Spectrometry-Based Approach for Mapping Protein Subcellular Localization Reveals the Spatial Proteome of Mouse Primary Neurons
Source: Cell Rep. 2017 Sep 12;20(11):2706–18. doi: 10.1016/j.celrep.2017.08.063 (PMC5775508; doi:10.1016/j.celrep.2017.08.063)
Supplement: Document S1. Supplemental Experimental Procedures, Figures S1–S6, and Tables S2 and S5 [file mmc1.pdf]

Cell Reports, Volume *20*

Supplemental Information

A Mass Spectrometry-Based Approach for Mapping Protein Subcellular Localization Reveals the Spatial Proteome of Mouse Primary Neurons

Daniel N. Itzhak, Colin Davies, Stefka Tyanova, Archana Mishra, James Williamson, Robin Antrobus, Jürgen Cox, Michael P. Weekes, and Georg H.H. Borner

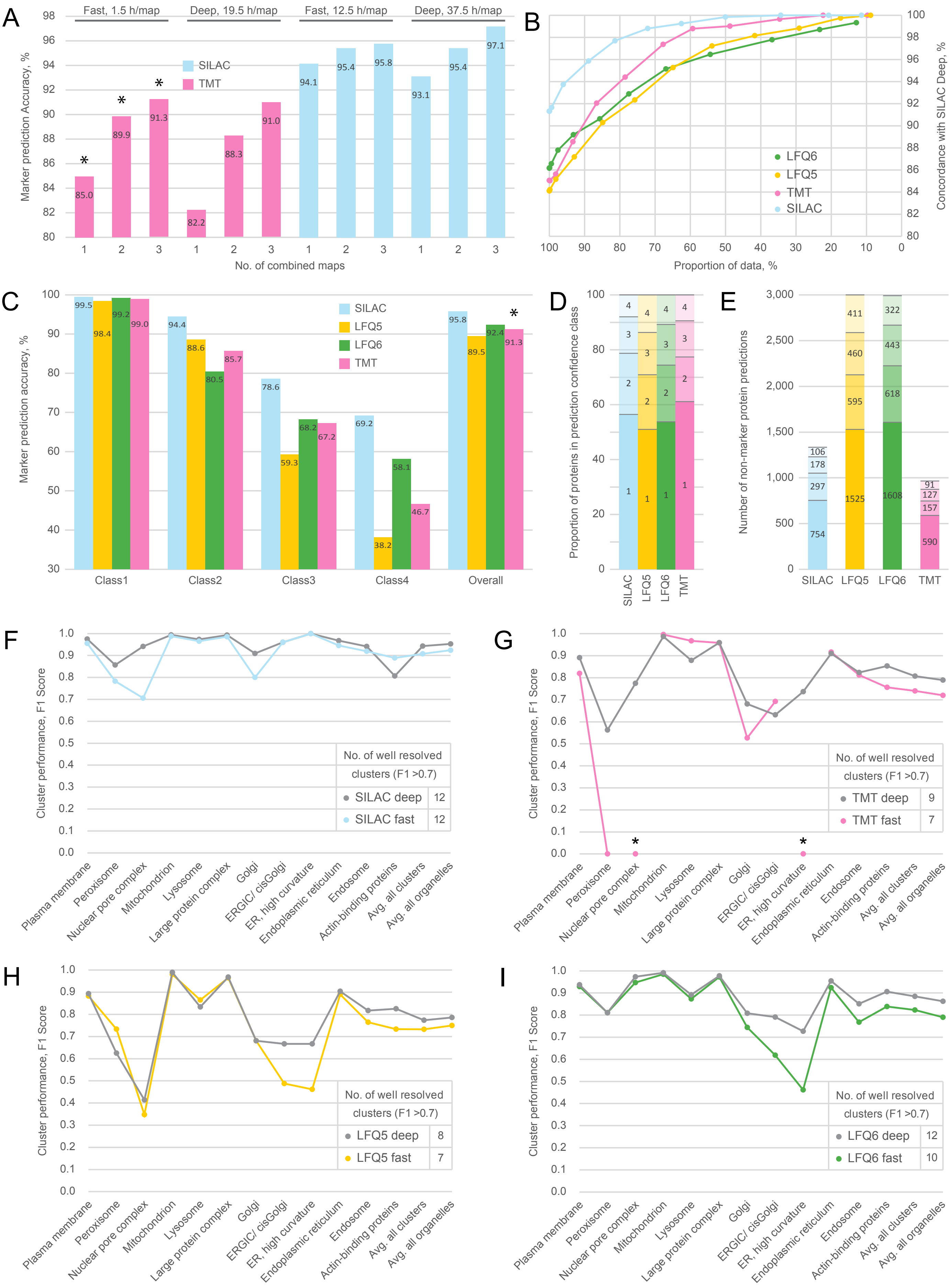
A
Fast, 1.5 h/map
Deep, 19.5 h/map
Fast, 12.5 h/map
Deep, 37.5 h/map
SILAC
TMT
Marker prediction Accuracy, %
No. of combined maps
85.0
89.9
91.3
82.2
88.3
91.0
94.1
95.4
95.8
93.1
95.4
97.1
*
B
LFQ6
LFQ5
TMT
SILAC
Proportion of data, %
Concordance with SILAC Deep, %
C
SILAC
LFQ5
LFQ6
TMT
Marker prediction accuracy, %
99.5
98.4
99.2
99.0
94.4
88.6
80.5
85.7
78.6
59.3
68.2
67.2
69.2
38.2
58.1
46.7
95.8
89.5
92.4
91.3
Class1
Class2
Class3
Class4
Overall
D
Proportion of proteins in prediction confidence class
SILAC
LFQ5
LFQ6
TMT
E
Number of non-marker protein predictions
106
178
297
754
411
460
595
1525
322
443
618
1608
91
127
157
590
SILAC
LFQ5
LFQ6
TMT
F
Cluster performance, F1 Score
No. of well resolved clusters (F1 >0.7)
SILAC deep 12
SILAC fast 12
G
Cluster performance, F1 Score
No. of well resolved clusters (F1 >0.7)
TMT deep 9
TMT fast 7
H
Cluster performance, F1 Score
No. of well resolved clusters (F1 >0.7)
LFQ5 deep 8
LFQ5 fast 7
I
Cluster performance, F1 Score
No. of well resolved clusters (F1 >0.7)
LFQ6 deep 12
LFQ6 fast 10
Plasma membrane
Peroxisome
Nuclear pore complex
Mitochondrion
Lysosome
Large protein complex
Golgi
ERGIC/ cisGolgi
ER, high curvature
Endoplasmic reticulum
Endosome
Actin-binding proteins
Avg. all clusters
Avg. all organelles

Figure S1. Further performance analyses of organellar maps generated with TMT, LFQ and SILAC quantification strategies. Related to Figure 2. A) Global prediction accuracy of maps made with TMT and SILAC workflows. Accuracy is the proportion of correctly predicted marker proteins; combining maps for classification increases prediction accuracy. *Please note that for TMT fast, classification was only performed on 10 clusters (see also G)). B) Concordance analysis for predictions made with the 'fast' MS protocol (three replicates combined). Predictions from the SILAC, TMT, and LFQ fast MS workflows were compared to the predictions obtained with the SILAC deep MS workflow, as in Figure 2K. C-E) Stratification of organellar predictions into confidence classes, as in Figure 2H-J, but for data obtained with the fast MS workflow. F-I): Detailed performance profiles of maps made with SILAC, LFQ5/6, and TMT, three replicates combined, comparing fast and deep MS protocols for each method. F1 scores are the harmonic mean of precision and recall of marker protein predictions for individual compartments. Clusters with F1 scores >0.7 have high predictive value.* For TMT fast maps, 'Nuclear pore complex' and 'ER high curvature' clusters had too few proteins to make SVM models, and were not included in the classification analysis; average F1 scores were hence calculated for only 10 clusters in this case. Peroxisomes were included, but not resolved (F1 = 0).

Fast Map Protocol

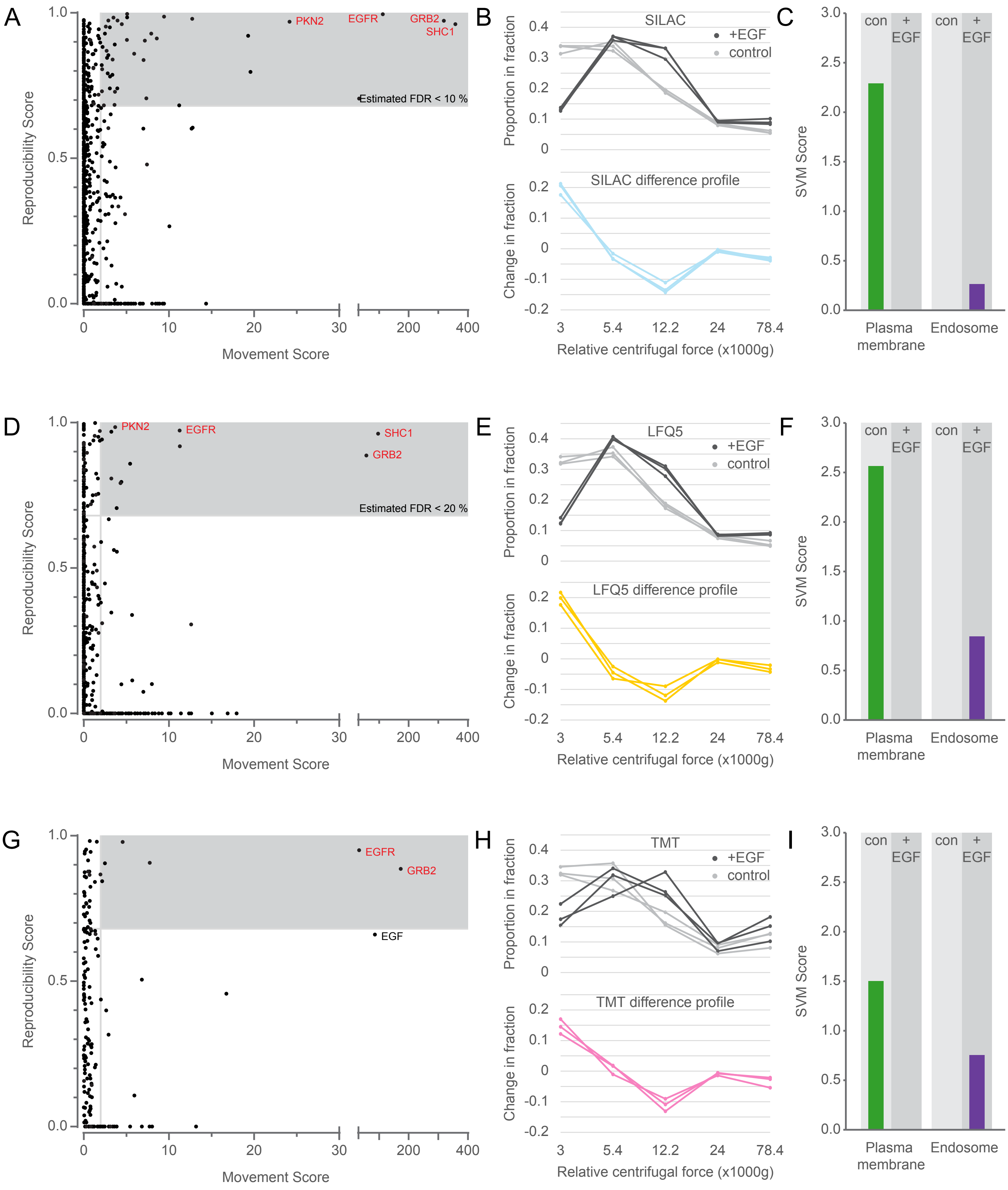

Figure S2. Assessment of Dynamic Organellar Maps with different quantification strategies using the fast MS protocol. Related to Figure 3. This figure is laid out exactly as Figure 3, except that data were generated with the fast MS protocol, which has reduced depth. Nonetheless, movement of EGFR from the plasma membrane to the endosome is captured with all methods.

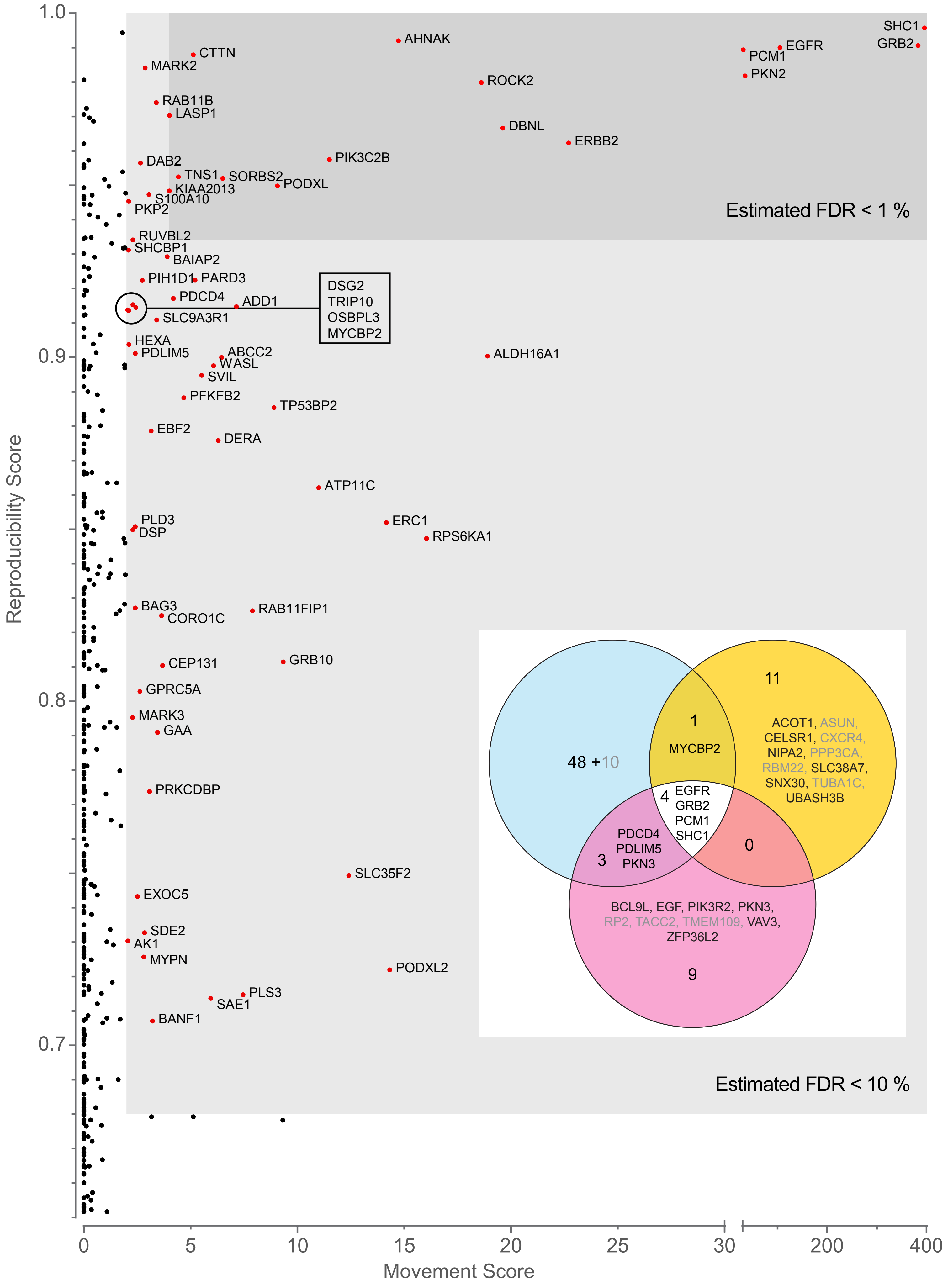

Reproducibility Score
Movement Score
1.0
0.9
0.8
0.7
0
5
10
15
20
25
30
200
400
Estimated FDR < 1 %
Estimated FDR < 10 %
SHC1
GRB2
EGFR
PCM1
PKN2
AHNAK
CTTN
MARK2
ROCK2
RAB11B
LASP1
DBNL
ERBB2
DAB2
PIK3C2B
TNS1
SORBS2
PODXL
KIAA2013
S100A10
PKP2
RUVBL2
SHCBP1
BAIAP2
PIH1D1
PARD3
PDCD4
ADD1
SLC9A3R1
DSG2
TRIP10
OSBPL3
MYCBP2
HEXA
PDLIM5
ABCC2
WASL
SVIL
ALDH16A1
PFKFB2
TP53BP2
EBF2
DERA
ATP11C
PLD3
DSP
ERC1
RPS6KA1
BAG3
CORO1C
RAB11FIP1
CEP131
GRB10
GPRC5A
MARK3
GAA
PRKCDBP
SLC35F2
EXOC5
SDE2
AK1
MYPN
PODXL2
PLS3
SAE1
BANF1
48 +10
1
MYCBP2
11
ACOT1, ASUN,
CELSR1, CXCR4,
NIPA2, PPP3CA,
RBM22, SLC38A7,
SNX30, TUBA1C,
UBASH3B
4
EGFR
GRB2
PCM1
SHC1
3
PDCD4
PDLIM5
PKN3
0
BCL9L, EGF, PIK3R2, PKN3,
RP2, TACC2, TMEM109, VAV3,
ZFP36L2
9

Figure S3. Detailed view of EGF translocation analysis. Related to Figure 3. MR plot reproduced from Figure 3A with all 66 proteins identified as moving in response to EGF, based on the SILAC deep analysis, annotated with their gene names. Two shaded areas indicate two estimated FDRs based on a mock-control experiment; in the darker shaded area, less than 1% false positives are expected, while in the lighter shaded area <10% of false positives are expected. Inset, Venn diagram showing the overlap of moving proteins detected with the three methods (deep protocol), corresponding to Figure 3A, D, G; SILAC, blue, LFQ, yellow, and TMT, pink. Proteins that were present in the SILAC dataset but were identified as hits only in the TMT or LFQ method are coloured grey. Conversely, proteins not present in the SILAC dataset but identified as hits only in TMT or LFQ are shown in black. Furthermore, there were 8 proteins among the SILAC hits that were not present in the TMT dataset, and a further 2 proteins absent from the LFQ dataset, denoted by +10, in grey.

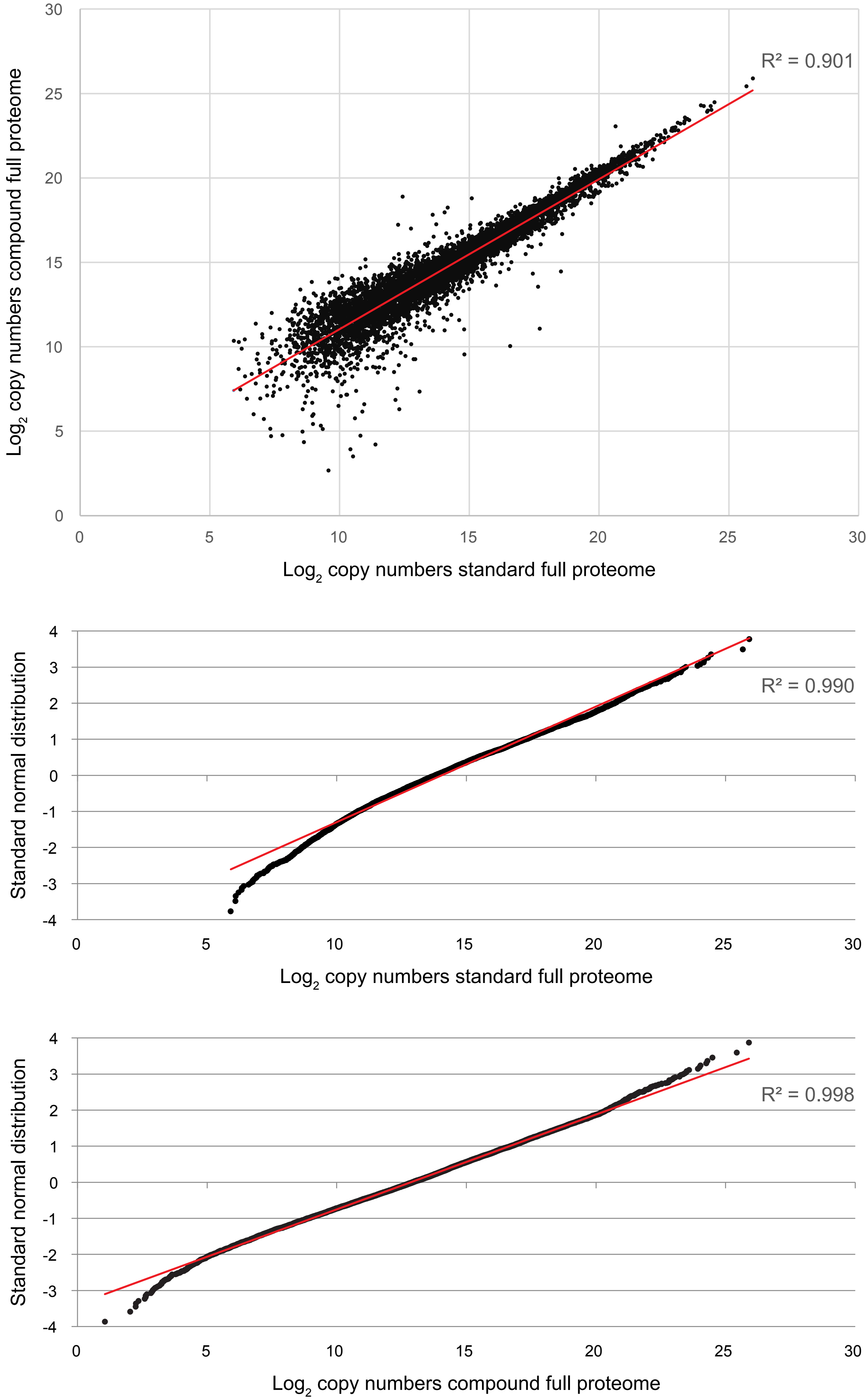
R² = 0.901
Log2 copy numbers compound full proteome
Log2 copy numbers standard full proteome
R² = 0.990
Standard normal distribution
Log2 copy numbers standard full proteome
R² = 0.998
Standard normal distribution
Log2 copy numbers compound full proteome

Figure S4. Deriving protein copy numbers/cell with two different strategies. Related to Figure 5. Protein copy numbers per cell were calculated with the Proteomic Ruler approach (Wisniewski et al., 2014). This requires determination of a whole cell proteome by mass spectrometry as input. Here, two approaches were compared: the 'standard' full proteome measured from whole cell lysates, and a 'compound' full proteome obtained by combining the mass spectrometric measurements of seven subcellular fractions (Figure 5A, and Supplemental Experimental Procedures). This figure shows that both yield very consistent results, but the compound data has greater depth. A) Copy numbers of proteins common to both datasets show high levels of correlation (R=0.949, R^2=0.9), and very similar absolute values. B, C) Distribution of copy numbers obtained with the two methods. Data were log transformed, and analysed by QQ plots. Perfectly normally distributed data would follow a straight line. As can be seen, both datasets show excellent correlation with theoretical standard normally distributed data (R^2>0.99). Copy numbers from compound proteomes in C) have an extended dynamic range at the lower end.

Table S2, related to Figure 3: Literature search results on proteins that move significantly in response to stimulation with EGF, as annotated in Figure S3, related to Figure 3.

Canonical ID	Gene name	M SCORE	R SCORE	Supporting reference for involvement in EGF signalling
P29353	SHC1	395.9	0.996	(Sakaguchi et al., 1998)
Q09666	AHNAK	14.7	0.992	No previous link identified
P62993	GRB2	382.9	0.991	(Lowenstein et al., 1992)
P00533	EGFR	105.4	0.990	(Sherrill and Kyte, 1996)
Q15154	PCM1	31.3	0.989	(Akimov et al., 2011)
Q14247	CTTN	5.1	0.988	(Mader et al., 2011)
Q7KZI7	MARK2	2.9	0.984	No previous link identified
Q16513	PKN2	35.0	0.982	(Vincent and Settleman, 1997)*
O75116	ROCK2	18.6	0.980	(Julian and Olson, 2014)
Q15907	RAB11B	3.4	0.974	(Watanuki et al., 2014)
Q14847	LASP1	4.0	0.970	No previous link identified
Q9UJU6	DBNL	19.6	0.967	No previous link identified
P04626	ERBB2	22.7	0.962	(Karunagaran et al., 1996)
O00750	PIK3C2B	11.5	0.957	(Wheeler and Domin, 2001)
P98082	DAB2	2.7	0.956	(Eskova et al., 2014)
Q9HBL0	TNS1	4.4	0.952	(Pai et al., 2001)
O94875	SORBS2	6.5	0.952	(Soubeyran et al., 2003)
O00592	PODXL	9.1	0.950	(Larsson et al., 2016)
Q8IYS2	KIAA2013	4.0	0.948	No previous link identified
P60903	S100A10	3.0	0.947	No previous link identified
Q99959	PKP2	2.1	0.945	(Arimoto et al., 2014)
Q9Y230	RUVBL2	2.3	0.934	(Kozik et al., 2013)
Q8NEM2	SHCBP1	2.1	0.931	(Schmandt et al., 1999)
Q9UQB8	BAIAP2	3.9	0.929	(Lewis-Saravalli et al., 2013)
Q8TEW0	PARD3	5.2	0.922	(Wang et al., 2006)
Q9NWS0	PIH1D1	2.7	0.922	(Fan et al., 2009; Kamano et al., 2013)
Q53EL6	PDCD4	4.2	0.917	(Matsuhashi et al., 2014)
Q14126	DSG2	2.3	0.915	(Klessner et al., 2009)
P35611	ADD1	7.1	0.915	(Fukata et al., 1999)
Q15642	TRIP10	2.4	0.914	(Hu et al., 2009)
Q9H4L5	OSBPL3	2.1	0.914	No previous link identified
O75592	MYCBP2	2.1	0.914	(Holland et al., 2011)
O14745	SLC9A3R1	3.4	0.911	Uniprot*
P06865	HEXA	2.1	0.904	No previous link identified
Q96HC4	PDLIM5	2.4	0.901	Uniprot
Q8IZ83	ALDH16A1	18.9	0.900	No previous link identified
Q92887	ABCC2	6.4	0.900	No previous link identified
O00401	WASL	6.1	0.898	(Galovic et al., 2011)
O95425	SVIL	5.5	0.895	(Fang et al., 2010)
O60825	PFKFB2	4.7	0.888	(Novellasdemunt et al., 2013)
Q13625	TP53BP2	8.9	0.885	(Liu et al., 2015)
Q9HAK2	EBF2	3.2	0.879	No previous link identified
Q9Y315	DERA	6.3	0.876	No previous link identified
Q8NB49	ATP11C	11.0	0.862	Uniprot
Q8IUD2	ERC1	14.2	0.852	No previous link identified
Q8IV08	PLD3	2.4	0.851	(Tong et al., 2014)
P15924	DSP	2.3	0.850	(Lorch et al., 2004)
Q15418	RPS6KA1	16.1	0.847	(Zhang et al., 2015)
O95817	BAG3	2.4	0.827	No previous link identified
Q6WKZ4	RAB11FIP1	7.9	0.826	(Xu et al., 2016)

Q9ULV4	CORO1C	3.6	0.825	(Hosseinibarkooie et al., 2016)
Q13322	GRB10	9.3	0.811	(He et al., 1998)
Q9UPN4	CEP131	3.7	0.810	No previous link identified
Q8NFJ5	GPRC5A	2.6	0.803	(Zhong et al., 2015)
P27448	MARK3	2.3	0.795	No previous link identified
P10253	GAA	3.5	0.791	No previous link identified
Q969G5	PRKCDBP	3.1	0.774	Uniprot
Q8IXU6	SLC35F2	12.4	0.749	No previous link identified
O00471	EXOC5	2.5	0.743	(Fogelgren et al., 2014)
Q6IQ49	SDE2	2.8	0.733	No previous link identified
P00568	AK1	2.1	0.730	No previous link identified
Q86TC9	MYPN	2.8	0.726	No previous link identified
Q9NZ53	PODXL2	14.3	0.722	No previous link identified
P13797	PLS3	7.5	0.715	(Hosseinibarkooie et al., 2016)
Q9UBE0	SAE1	5.9	0.714	No previous link identified
O75531	BANF1	3.2	0.707	No previous link identified

A primary literature search was conducted to identify links between EGF receptor signalling and the identified hits; references for these links are provided where possible. This search was not exhaustive; some links may have been missed, and apologies are made to those whose work was not cited. *a link between this protein and EGFR was not identified, but a link between this and a second hit in the list, which is linked to EGFR signalling, was identified.

Table S5, related to Figure 5: Comparison of spatial proteomic profiling methods

**Method**	**Dynamic Organellar Maps**		**hyperLOPIT**	**PCP**	**Rat liver proteome**
Reference	Itzhak et al., 2016	This Study	Christoforou et al., 2016	Foster et al., 2006	Jadot et al., 2017
Organelle separation technique	Differential centrifugation		Density gradient centrifugation	Velocity gradient centrifugation	Combination of different centrifugation techniques
Quantification approach	Metabolic labelling (SILAC)	Label free quantification (LFQ) or Isobaric labelling (TMT)	Isobaric labelling	Label free quantification	Isobaric labelling
Instrumentation	Q Exactive HF	Q Exactive HF (for LFQ) Orbitrap Fusion/Lumos (for TMT)	Orbitrap Fusion	Orbitrap*	Orbitrap Velos
Starting material per map	1E7 HeLa cells	1 -2 mg protein (mouse neurons, for LFQ)	1E8 mouse embryonic stem cells	1 mouse liver	2 rat livers
Number of MS analysis runs per map	24	7 (LFQ)	24	32	>200
Number of mapped proteins	>8,700 (from 6 replicates)	>8,000 (from 5 replicates)	>5,500 (from 2 replicates)	>1,400*	>6,000 (from 4 replicates)
Strengths of the method	High organellar resolution<br>High reproducibility allows comparative applications<br>Quantitative organellar modelling		High organellar resolution<br>Comparative applications (Jean Beltran et al., 2016)	Assignment of one protein to multiple organellar compartments possible	Assignment of one protein to multiple organellar compartments, with quantification

*The performance of mass spectrometers has substantially improved since the publication of PCP in 2006; a repeat analysis with current MS instruments would very likely show a depth of analysis comparable to the other methods.

Supplemental Experimental Procedures

Analysed samples - Overview

In this study, we prepared maps from several new samples, but also re-analysed several previously generated samples (Itzhak et al., 2016), either with new labelling and new mass spectrometry (MS), or new processing (summarised in the table below). All analyses were performed with fast (short MS run time) and deep (extensive MS run time) protocols.

The aim of the study was two-fold: firstly, to develop and evaluate workflows for Label-Free Quantification (LFQ) and Tandem Mass Tagging (TMT)-based organellar maps; and secondly, to compare the performance of different labelling strategies for comparative Dynamic Organellar Maps. To establish the label-free workflow, six maps were prepared from fresh samples (HeLa untreated). To ensure a fair comparison of SILAC, TMT, and LFQ approaches for dynamic applications, maps were prepared from the exact same set of samples, generated previously (HeLa untreated vs HeLa stimulated with EGF (Itzhak et al., 2016)). This published set was SILAC labelled, and analysed in a 'fast' format (Itzhak et al., 2016). Here, a 'deep' analysis of the same samples was added (i.e. a new in-depth MS analysis). For TMT maps, SILAC light subfractions were labelled with TMT reagent, and analysed by MS. For dynamic LFQ maps, SILAC RAW files were re-processed in MaxQuant software, ignoring the heavy channel, to simulate a label-free experiment (see 'Processing of mass spectrometry data' below for details).

Samples analysed in this study

Sample ID	Sample, treatment (Reference), original labelling	Labelling for this study	Mass spec method	Reprocessed or New Mass spec analysis	Used for
1	3 x HeLa, untreated (Itzhak et al., 2016), SILAC light subfractions and heavy reference fraction	SILAC	Deep	Reprocessed	SILAC maps, static
2	3 x HeLa, untreated (Itzhak et al., 2016), SILAC light subfractions and heavy reference fraction	SILAC<br>SILAC<br>SILAC	Fast<br>Fast<br>Deep	Reprocessed<br>Reprocessed<br>New mass spec	SILAC maps, static<br>SILAC maps, dynamic<br>SILAC maps, dynamic
3	3 x HeLa, 20 min EGF treated (Itzhak et al., 2016), SILAC light subfractions and heavy reference fraction	SILAC<br>SILAC	Fast<br>Deep	Reprocessed<br>New mass spec	SILAC maps, dynamic<br>SILAC maps, dynamic
1	6 x HeLa, untreated (Itzhak et al., 2016), SILAC light subfractions and heavy reference fraction	SILAC	Deep	Reprocessed	SILAC maps, 'mock' dynamic (for FDR control)
4	6 x HeLa, untreated (this study), no label (3 of them selected for additional deep analysis)	Label free<br>Label free	Fast<br>Deep	New mass spec<br>New mass spec	LFQ maps, static<br>LFQ maps, static
2	3 x HeLa, untreated (Itzhak et al., 2016), SILAC light subfractions and heavy reference fraction	Process as label free<br>Process as label free	Fast<br>Deep	Reprocessed<br>New mass spec	LFQ maps, dynamic<br>LFQ maps, dynamic
3	3 x HeLa, 20 min EGF treated (Itzhak et al., 2016), SILAC light subfractions and heavy reference fraction	Process as label free<br>Process as label free	Fast<br>Deep	Reprocessed<br>New mass spec	LFQ maps, dynamic<br>LFQ maps, dynamic
1	6 x HeLa, untreated (Itzhak et al., 2016), SILAC light subfractions and heavy reference fraction	Process as label free	Deep	Reprocessed	LFQ maps, 'mock' dynamic (for FDR control)

2	3 x HeLa, untreated (Itzhak et al., 2016), SILAC light subfractions only	TMT 10-plex	Fast	New mass spec	TMT maps, static and dynamic
		TMT 10-plex	Deep	New mass spec	TMT maps, static and dynamic
3	3 x HeLa, 20 min EGF treated (Itzhak et al., 2016), SILAC light subfractions only	TMT 10-plex	Fast	New mass spec	TMT maps, dynamic
		TMT 10-plex	Deep	New mass spec	TMT maps, dynamic
5	5 x mouse neurons, acutely isolated, no label (this study)	Label free	Fast	New mass spec	LFQ maps of mouse neurons

Cell Culture

For the generation of label-free organellar maps, HeLa cell cultures were maintained as described (Itzhak et al., 2016), but using regular Dulbecco's Modified Eagle's Medium (DMEM) and fetal calf serum (instead of SILAC labelling medium and dialysed fetal calf serum).

Subcellular fractionation procedure for label-free organellar maps

Cell lysis and subcellular fractionation were identical to our previously reported protocol (Itzhak et al., 2016), but omitting any steps relating to the SILAC heavy labelled reference sample. Each map was prepared from a single ~70% confluent 15 cm dish of HeLa cells.

Cortical neuron preparation

Mice (C57BL/6 background) were housed in an SPF facility with 12:12 h light/dark cycle and food/water available ad libitum. All animal experiments were performed in compliance with institutional policies approved by the government of upper Bavaria. For preparation of cortical neurons, the procedure described in Meberg and Miller (2003) was adapted. One E15 pregnant mice was sacrificed by cervical dislocation, the uterus was removed from the abdominal cavity and placed into a 10 cm sterile petri dish containing cold Hanks' balanced salt solution (HBSS) on ice. Each fetus was isolated, heads of embryos were quickly cut, brains were removed from the skull and immersed in ice cold HBSS. Subsequently, cortical hemispheres were dissected and meninges were removed under a stereo-microscope. For each sample, cortical tissue from typically six to seven embryos (from one litter) was cut into smaller pieces, transferred to 15 ml sterile tube and treated with 0.25% trypsin containing 1 mM EDTA for 20 minutes at 37°C. The enzymatic reaction was stopped by removing the supernatant and washing the tissue twice with Neurobasal medium (Invitrogen) containing 5% Fetal Bovine Serum. The tissue was resuspended in 2 ml medium and triturated 10 strokes with the tip of a Pasteur pipette. Single cell suspension was achieved by triturating an additional 10 strokes with a fire-polished pipette. Cells were spun at 180 x g, the supernatant was removed and the cell pellet was stored on ice till further use.

Please note that this preparation procedure yields fairly pure neuronal populations (Xu et al., 2012), since glial cells have not yet developed at stage E15 (Qian et al., 2000). Supporting this notion, we detected neuronal markers as highly abundant proteins in our complete neuron proteome (Table S4; eg Nestin, 91th abundance percentile; Tubb3, 98th abundance percentile), whereas markers of glial cells (eg GFAP (astrocyte marker) and Cldn11/Ops (oligodendrocyte marker)) were undetectable. Red blood cells were a very minor contaminant of the preparation (estimated at ca. 1.5%, based on levels of detected hemoglobin). Since the red blood cell proteome is mostly dominated by hemoglobin itself (Bryk and Wisniekswi, 2017), the contamination of the neuron proteome with other red blood cell proteins was considered negligible.

Owing to the necessary cell dissociation step, isolated neurons are prone to losing their neurites during the preparation procedure. Since cells can be cultured subsequently (Meberg and Miller, 2003), this does not appear to compromise cell viability. The axonal and dendritic parts of the plasma membrane (in addition to a proportion of cytosol) are hence lost to some extent, and will not be accounted for in the proteome. This is unavoidable for the analysis of acutely isolated neurons. Importantly, at this early stage of development, there is not extensive dendritic or axonal arborisation (Sciarretta and Minichiello, 2010), so the loss of neurites should account for a fairly small proportion of the total cellular material. The big advantage of using acutely isolated neurons is that

the cells have not been exposed to any culture conditions, and, with the above restrictions, should faithfully reflect neuronal composition as encountered in the brain.

Generation of organellar maps from neurons

In total, six independent neuron preparations were performed, on three separate days (two pregnant females/day). Isolated neurons were immediately processed for organellar mapping. For generation of Map 1, neurons from preparations 1&2 were pooled. For Maps 2-5, neurons prepared from a single litter were used. A typical prep corresponded to 1-2 mg of protein as starting material for subcellular fractionation.

Cells were resuspended in ice-cold PBS and centrifuged at 250 x g for 5 minutes. Cells were then resuspended in 5 ml homogenisation buffer (25 mM Tris pH 7.4, 50 mM Sucrose, 0.2 mM EGTA, 0.5 mM MgCl2) for 5 minutes. For Map 1, cells were lysed in a Dounce homogenizer (Sartorius, tight pestle; 15 strokes). For Maps 2-5, cells were passed through a cell cracker (Isobiotec) fitted with a 10 µm bore (5 passes) to achieve lysis. The remainder of the fractionation protocol followed the procedure previously described in detail for HeLa cells (Itzhak et al., 2016).

Sample preparation for mass spectrometry

SILAC and LFQ samples

Protein digestion, peptide cleanup and peptide fractionation of label free and SILAC based samples were performed as described (Itzhak et al., 2016).

TMT sample preparation

Protein fractions in SDS buffer (2.5% SDS, 50 mM Tris pH 8.1) were precipitated with 100% acetone and resuspended in 6M guanidine, 50 mM HEPES pH 8.5. Dithiothreitol (DTT) was added to a final concentration of 5 mM and samples were incubated for 20 minutes. Cysteines were alkylated with 14 mM iodoacetamide and incubated 20 minutes at room temperature in the dark. Excess iodoacetamide was quenched with DTT for 15 min. Samples were diluted with 200 mM HEPES pH 8.5 to 1.5 M Guanidine, followed by digestion at room temperature for 3 hr with LysC protease at a 1:100 protease-to-protein ratio. Following LysC digestion, trypsin was then added at a 1:100 protease-to-protein ratio followed by overnight incubation at 37°C. The reaction was quenched with 2% formic acid, subjected to C18 solid-phase extraction (Sep-Pak, Waters) and vacuum-centrifuged to near-dryness. TMT labelling was performed as previously described (Weekes et al., 2014). Briefly, desalted peptides were dissolved in 200 mM HEPES pH 8.5. Peptide concentration was measured by micro BCA (Pierce), and 25 µg of peptide labelled with TMT reagent at a final (AcN) concentration of 30% (v/v). Samples were labelled as follows: Control 3k (TMT 126); Control 5.4k (TMT 127N); Control 12.2k (TMT 127C); Control 24k (TMT 128N); Control 78.4k (TMT 128C); EGF-treated 3k (TMT 129N); EGF-treated 5.4k (TMT 129C); EGF-treated 12.2k (TMT 130N); EGF-treated 24k (TMT 130C); EGF-treated 78.4k (TMT 131). Following incubation at room temperature for 1 h, the reaction was quenched with hydroxylamine to a final concentration of 0.5% (v/v). TMT-labeled samples were combined at a 1:1:1:1:1:1:1:1:1:1 ratio. The sample was vacuum-centrifuged to near dryness and subjected to C18 solid-phase extraction (SPE) (Sep-Pak, Waters).

Off-line high pH reversed-phase (HpRP) peptide fractionation of TMT labelled samples (deep protocol)

TMT-labelled tryptic peptides were subjected to HpRP-HPLC fractionation using an Ultimate 3000 RSLC UHPLC system (Thermo Fisher Scientific) equipped with a 2.1 i.d x25 cm, 1.7 µm particle Kinetix Evo C18 column (Phenomenex). Mobile phase consisted of A: 3% AcN, B: AcN and C: 200 mM ammonium formate pH 10. Isocratic conditions were 90% A/10% C and C was maintained at 10% throughout the gradient elution. Separations were carried out at 45°C. After loading at 200 µL/minute for 5 minutes and ramping the flow rate to 400 µL/minute over 5 minutes the gradient elution proceed as follows: 0-19% B over 10 minutes (curve 3), 19-34% B over 14.25 minutes (curve 5), 34-50% B over 8.75 minutes (curve 5), followed by a 10 minutes wash at 90% B. UV absorbance was monitored at 280 nm and 15 s fractions were collected into 96 well microplates using the integrated fraction collector. Peptide containing fractions were then orthogonally recombined into 24

fractions and dried in a vacuum centrifuge and resuspended in 10 µL MS solvent (4% AcN / 5% formic acid (FA) prior to LC-MS3). 12 combined fractions were used for MS analysis of replicates 1 and 3. In replicate 2, a contaminant of unknown origin reduced the MS performance; hence, all 24 combined fractions were used for MS analysis.

Mass spectrometric (MS) analysis

SILAC and LFQ samples

Mass spectrometric analysis of SILAC and LFQ samples was performed as described (Itzhak et al., 2016). Briefly, peptides were loaded onto a 50-cm column with 75-µm inner diameter, packed in-house with 1.8-µm C18 particles (Dr Maisch GmbH, Germany), attached to an EASY-nLC 1000 (Thermo Fisher Scientific, Germany). Peptide separation was achieved with a binary buffer system consisting of 0.1% formic acid (buffer A), and 80% acetonitrile in 0.1% formic acid (buffer B), using a linear gradient of buffer B from 2% to 30% in 130 min followed by washout (ramping to 95% B in 5 min, constant at 95% B for 5 min, ramping down to 2% B in 5 min, constant at 2% B for 5 min), at a flow rate of 250 nl/min. The column was operated at 55°C. The LC was coupled to a Q Exactive HF Hybrid Quadrupole-Orbitrap mass spectrometer (Thermo Fisher Scientific, Germany). MS data were acquired using a data-dependent top 15 method. Survey scans were acquired at a resolution of 120,000, and HCD spectra at a resolution of 15,000. The dynamic exclusion of sequenced peptides was set to 30s.

TMT samples

For samples in the TMT workflow, mass spectrometry data for replicates 1 and 2 was acquired with an Orbitrap Lumos and replicate 3 with an Orbitrap Fusion (Thermo Fisher Scientific, San Jose, CA).

Mass spectrometric analysis of TMT samples with an Orbitrap Lumos

Samples were injected onto an Ultimate 3000 RSLC nano UHPLC equipped with a 300 µm i.d. x 5 mm Acclaim PepMap µ-Precolumn (Thermo Fisher Scientific) and a 75 µm i.d. x50 cm 2.1 µm particle Acclaim PepMap RSLC analytical column. Loading solvent was 0.1% FA, analytical solvent A: 0.1% FA and B: AcN + 0.1% FA. All separations are carried out at 55°C. Samples were loaded at 5 µL/minute for 5 minutes in loading solvent before beginning the analytical gradient. The following gradient was used: 3-7% B over 4 minutes, 7 – 37% B over 176 minutes, followed by a 10 minutes wash at 90% B and equilibration at 3% B for 5 minutes. Each analysis on the Orbitrap Lumos mass spectrometer (Thermo Fisher Scientific) used a MultiNotch MS3-based TMT method (McAlister et al., 2012, 2014). The following settings were used: MS1: 400-1200 Th, Quadrupole isolation, 120,000 Resolution, $2x10^5$ AGC target, 50 ms maximum injection time, ions injected for all parallisable time. MS2: Quadrupole isolation at an isolation width of m/z 0.7, CID fragmentation (NCE 35) with ion trap scanning out in turbo mode from m/z 120, $1.5x10^4$ AGC target, 120 ms maximum injection time, ions accumulated for all parallisable time in centroid mode. MS3: In Synchronous Precursor Selection mode the top 10 MS2 ions were selected for HCD fragmentation (NCE 65) and scanned out in the Orbitrap at 60,000 resolution with an AGC target of $1x10^5$ and a maximum accumulation time of 150 ms, ions were not accumulated for all parallelisable time. The entire MS/MS/MS cycle had a target time of 3 s. Dynamic exclusion was set to +/- 10 ppm for 70 s. MS2 fragmentation was trigged on precursors $5x10^3$ counts and above.

Mass spectrometric analysis of TMT samples with an Orbitrap Fusion

Samples were injected onto an Ultimate 3000 RSLC nano UHPLC equipped with a 300 µm i.d. x 5 mm Acclaim PepMap µ-Precolumn (Thermo Fisher Scientific) and a 75 µm i.d. x50 cm 2.1 µm particle Acclaim PepMap RSLC analytical column. Loading solvent was 0.1% TFA, analytical solvent A: 0.1% FA and B: AcN + 0.1% FA. All separations are carried out at 55°C. Samples were loaded at 10 µL/minute for 5 minutes in loading solvent before beginning the analytical gradient. The following gradient was used: 3-5.6% B over 4 minutes, 5.6 – 32% B over 162 minutes, followed by a 5 minute wash at 80% B and a 5 minute wash at 90% B and equilibration at 3% B for 5 minutes. Each analysis on the Orbitrap Lumos mass spectrometer (Thermo Fisher Scientific) used a MultiNotch MS3-based TMT method (McAlister et al., 2012, 2014). The following settings were used: MS1: 400-1400 Th, Quadrupole isolation, 120,000 Resolution, $2x10^5$ AGC target, 50 ms maximum injection time, ions injected for all parallisable time. MS2: Quadrupole isolation at an isolation width

of m/z 0.7, CID fragmentation (NCE 30) with ion trap scanning out in rapid mode from m/z 120, $1x10^4$ AGC target, 70 ms maximum injection time, ions accumulated for all parallisable time in centroid mode. MS3: in Synchronous Precursor Selection mode the top 10 MS2 ions were selected for HCD fragmentation (NCE 65) and scanned out in the Orbitrap at 50,000 resolution with an AGC target of $5x10^4$ and a maximum accumulation time of 150 ms, ions were not accumulated for all parallelisable time. The entire MS/MS/MS cycle had a target time of 3 s. Dynamic exclusion was set to +/- 10 ppm for 90 s. MS2 fragmentation was trigged on precursors $5x10^3$ counts and above.

Overview of MS measurement time requirements

Labelling method	MS protocol	Subcellular fractions/ map	Peptide fractions	Total samples for MS	MS run time/fraction (hours)	Total MS run time/map
SILAC	Fast	5	1	5	2.5	12.5
	Deep	5	3	15	2.5	37.5
LFQ5	Fast	5	1	5	2.5	12.5
	Deep	5	3	15	2.5	37.5
LFQ6	Fast	6	1	6	2.5	15
	Deep	6	3	18	2.5	45
TMT	Fast	5	1	1	3	1.5**
	Deep	5	12+1*	13	3	19.5**

* For TMT deep maps, the 12 peptide fractions were jointly processed with the single file from the corresponding fast map.

**TMT 10-plex allows running of two maps in one sample; hence, total MS run time is shown as half the actual run time.

Processing of mass spectrometry data

Raw files were processed with MaxQuant Version 1.5.5.2 (Cox and Mann, 2008; Tyanova et al., 2016a), using the human reference protein dataset downloaded from UniProt (SwissProt canonical and isoforms database). Default settings were used for all analyses, with the following exceptions:

For SILAC samples, multiplicity was set to 2, with Arg10 and Lys8 as heavy isotopes. The minimum ratio count was set to 1. Re-quantification was enabled. Matching between runs was enabled, to allow matching between equivalent fractions of replicates only.

For unlabelled samples, LFQ quantification (Cox et al., 2014) was selected, with a minimum peptide count of 1. Matching between equivalent fractions was enabled, as for SILAC samples.

For SILAC samples processed to simulate a label free experiment, multiplicity was set to 1 (SILAC light channel detected only). Matching between equivalent fractions was enabled. Please note that this procedure will underestimate the performance of LFQ dynamic maps, since a large proportion of the MS^2 peptide sequencing will be performed on SILAC heavy peptides, which do not contribute the quantification of the light peptides. This substantially reduces the sequencing depth and oversampling rate compared to a genuine single channel MS analysis; peptide IDs are reduced by about 30-40%.

For TMT labelled samples, batch-specific correction factors were configured in the modifications tab in MaxQuant (TMT 10-plex lot no. QK226224). Sample type was set to ‘reporter ion MS3’ with all 10-plex TMT labels selected, for both Lysine and N-termini. Matching between fractions was not activated. For deep TMT maps, the 12 fractions obtained with high pH fractionation were processed jointly with the single fraction from the corresponding fast map. In replicate 2, a contaminant of unknown origin reduced the MS performance; hence, all 24 high pH fractions and the single fast map file were jointly analysed. Nevertheless, the performance of replicate 2 remained substantially lower (4959 complete profiles). The depth we report for replicates 1 and 3 (6059 and 6699 profiles, respectively), obtained with 12+1 fractions, is therefore likely to be representative of the performance.

For mouse neuron data, raw files were processed with MaxQuant Version 1.5.4.3 (Cox and Mann, 2008; Tyanova et al., 2016a), using the mouse reference protein dataset downloaded from UniProt (SwissProt canonical and isoforms database). Default settings were used for all analyses, with the following exceptions: LFQ quantification (Cox et al., 2014) was selected, with a minimum peptide count of 1. Since the mouse neuron maps were prepared in three batches (one map in batch 1, two maps each in batches 2 and 3), matching between runs was enabled for equivalent and adjacent fractions within each batch only.

Filtering and transformation of proteomic data

The primary output from MaxQuant is the 'protein groups' file, listing protein identifications and the quantifications across the (five or six) subfractions. For all datasets, matches to the reverse database, proteins identified only with modified peptides, and common contaminants were removed. Further filtering was tailored to each labelling strategy, to obtain high quality datasets for further analysis.

For SILAC, profile filtering was always performed at the level of individual maps. Each map consisted of five ratio quantifications. For each fraction, SILAC ratios were linearly normalized by division through the fraction median. Individual ratios were retained if they were based on more than two quantification events, or on two quantification events where the ratio variability did not exceed 31%. Only proteins with a complete set of five ratios were retained. These SILAC ratio profiles were used for organellar assignments (SVM predictions, see below). For a direct comparison of SILAC profiles with TMT and LFQ profiles, further (0 to 1) normalization of profiles was required. For each protein, inverted SILAC ratios (Light/Heavy) were divided buy their sum.

For static LFQ maps, profile filtering was performed at the level of individual maps. Each map consisted of six fractions with LFQ intensities. MaxLFQ intensities are already globally normalized, and need no further normalization correction. Owing to the high dynamic range of label-free quantification, profiles with some missing values could be tolerated. Two stringency filters were applied: first, only proteins with LFQ intensities in at least three out of the six fractions were retained; and second, the MS/MS count summed over all six fractions had to be at least 12 (i.e. two per fraction on average). For each protein, LFQ intensities were normalized to the sum of LFQ intensities across the six fractions, yielding a six-data point profile of relative intensities (summing to one). These profiles were used for the generation of 6-fraction LFQ organellar maps.

To evaluate the performance of 5 fraction LFQ maps, the 1K fraction was removed from the dataset, and the remaining five data points were re-normalised to their sum. Thus, 5 and 6 fraction LFQ maps contain the exact same sets of proteins, permitting a fair comparison of the contribution made by the sixth fraction.

For dynamic LFQ maps (based on reprocessed SILAC data), only 5-data point profiles were available. These were filtered for a minimum of four consecutive LFQ values per protein and map. The dynamic LFQ experiment consisted of six maps (three controls and three +EGF treatment). For determining the MS/MS counts filter, MS/MS events were summed across all six maps (i.e. across 30 fractions). Only proteins with 60 or more MS/MS events in total were retained in the dataset. This alternative filtering strategy greatly enhances the scope of the MR plot translocation analysis, as it allows the inclusion of proteins that are abundant under one of the tested conditions, but not the other.

Each TMT labelled map consisted of five fractions with corrected reporter intensities. To account for unequal peptide loading in each fraction, a set of correction factors were determined by calculating the total intensity of each fraction divided by the fraction with the lowest summed intensity. Each value in a fraction was then divided by its own correction factor. Each TMT 10-plex experiment contained two maps, but these were treated separately for normalisation. For static TMT maps, proteins with a minimum median reporter intensity count of 1 across the five fractions were retained. For each protein, TMT intensities were then normalized to the sum of intensities within a map, yielding a five-data point profile (summing to one).

For dynamic TMT maps, this filtering was applied per experiment (i.e. the minimum median intensity count had to be 1 across ten fractions). As with the dynamic LFQ maps, this enhances the depth of the MR plot analysis.

Please note that for dynamic SILAC, TMT and LFQ maps, profiles were weighted with fraction yields (percent of protein recovered in a given subcellular fraction, relative to total yield) prior to 0-1 normalisation (Itzhak et al., 2016). While this step is recommended, it is not absolutely required.

Datasets for organellar predictions

To evaluate the suitability of different labelling strategies for making static organellar maps, performance was assessed at the level of individual maps, as well as for combinations of multiple maps.

To combine maps for classification, the set of proteins common to all maps was determined. Combining maps reduces the depth compared to the individual maps, but enhances classification accuracy. Support vector machine based classification (see below) was then performed on the total data available for each protein (e.g. 15 data points for a combination of 3 SILAC maps), resulting in one set of organellar prediction scores (regardless of how many maps were combined).

For LFQ, six individual fast maps were generated and assessed individually. The two lowest performing maps were then combined, for a joint output, followed by adding the third-lowest performing map and so on, up to a combination of all six maps. In all cases, only the proteins common to all six datasets were analysed.

An equivalent analysis was then repeated with three deep LFQ maps. These maps were generated from the same samples used for three of the fast maps, but with much more extensive MS analysis.

For SILAC, three fast maps and three deep maps were analysed likewise.

The TMT data had a different structure, as the 10-plexing allowed the concomitant generation of two (2 x 5 fraction) maps in one sample. Here, a control map was always multiplexed with its cognate EGF treatment map. For the evaluation of TMT static map performance, only fractions of the control map were processed as standard (five fraction) maps. Thus, three fast and three deep (untreated) TMT maps were analysed.

Based on the analysis presented in this study, we recommend the generation of three replicate maps, both for static and dynamic applications. Hence, map performance of all labelling approaches and MS protocols (fast vs deep) was compared for a combination of three maps (Figure 2, Figure 4, Figure S1). For LFQ fast analysis, we selected three medium performing maps from our set of six available maps, and jointly reprocessed them in MaxQuant. To combine maps, only proteins with high quality profiles in each map were selected for making organellar predictions. These six combination datasets were then assessed for prediction performance, depth, and concordance.

Organellar marker sets

For map annotation and supervised learning, we used our previously published set of 1,076 organellar markers from 12 different subcellular compartments (Itzhak et al., 2016). For SILAC (deep), TMT (deep), LFQ (deep) and LFQ (fast) maps, sets of around 1,000 markers were matched in each case. For SILAC (fast) and TMT (fast) analyses, map depth was considerably lower, and only 801 and 572 markers were matched, respectively. The minimum size for a cluster was seven members. In the case of TMT (fast), two clusters were too small, and hence excluded from the supervised learning.

For the mouse neuron maps the same set was used, matching markers by gene names (834 proteins matched).

Generation of organellar maps

For organellar assignments, we used the machine learning approach described in Itzhak et al. (2016), as implemented in Perseus software (Tyanova et al., 2016b). Briefly, organellar makers were matched to each map. A support vector machine (SVM)-algorithm (with a radial basis function kernel) was trained on the marker proteins, with cross-validation to prevent overfitting. Supervised learning was then performed with the optimized parameters, using full (leave-one-out) cross-validation for performance assessment.

The analysis provides two outputs: firstly, a misclassification table (or ‘confusion matrix’), listing which marker proteins had been assigned (correctly or incorrectly) to which compartment; and secondly, a prediction table, in which proteins receive prediction scores for each of the 12 compartments. Each protein is then assigned to the compartment for which it received the highest score. The sum of all these organellar assignments constitutes an organellar map.

Prediction scores are a measure of how confidently a protein is assigned to a compartment. Classification by SVMs defines (non-linear) maximum-margin boundaries between clusters; the distance from a cluster to the boundary is set to 1. Predictions with scores >1 therefore fall within the space defined by the markers; predictions with scores between 0 and 1 fall between the marker cluster and the boundary to markers from other clusters; and predictions with scores <0 are beyond the boundary. A prediction with a score <0 can still be correct, but less likely so. We therefore stratified our predictions into four confidence classes:

Prediction confidence classes

SVM score	Confidence	Class
>1	High	1
0.5-1	Medium	2
0-0.5	Low	3
<0	Very Low	4

Please note that in our previous study (Itzhak et al., 2016), we used a more complex scoring system, since the complete output from six individual maps was combined (and not just the overlapping set). For single maps, or for a combination of maps using the overlapping set of proteins as in the present study, this new scheme is recommended, as it allows a more straightforward interpretation of the scores and confidence classes, and is also directly comparable between sets of maps.

Performance evaluation of organellar maps

A pre-defined set of ca. 1000 bona fide markers of 12 different subcellular localisations/organelles (Itzhak et al., eLife 2016) was used to annotate any new map dataset. Support vector machines were optimized with the marker proteins, to define boundaries between the organellar clusters (see above, Generation of organellar maps). The quality of the final SVM model was evaluated with full leave-one-out cross validation (eg when there were 1000 marker proteins, 1000 different SVM models were built, each with only 999 proteins; in each case it was checked if the missing protein was correctly predicted, to simulate the application of the model to non-marker data). The average proportion of correctly predicted marker proteins was then assessed, both globally, and by cluster. This serves as an estimate of the prediction accuracy for the remainder of the data, which are not marker proteins.

The following metrics were calculated to assess map performance:

1. Marker prediction accuracy was calculated as the fraction of all correctly predicted markers divided by the total number of markers in the set. This metric gives more weight to larger protein clusters (such as mitochondria, plasma membrane and ER), whose prediction accuracy will dominate the global accuracy.
2. To evaluate the prediction performance for individual clusters, we calculated recall and precision. Recall is the proportion of markers correctly assigned to this cluster (i.e. True Positives/(True Positives + False Negatives)). Precision is the number of markers correctly assigned to a cluster, relative to the number of all markers assigned to the cluster (i.e. True Positives/ (True Positives + False Positives). Both metrics can be combined into an ‘F1 score’ by calculating their harmonic mean. F1 Scores range from 0-1. Unless both metrics have the same value, the harmonic mean is always lower than the arithmetic mean, thus ensuring that only combinations of high precision and recall values can achieve high F1 scores. For example, a cluster with perfect precision (1) but poor recall (0.2) would get a low F1 score of 0.33. Plotting F1 scores for all map clusters (as in Figure 2G) reveals particular strengths and weaknesses of a map; in our experience, clusters with scores >0.7 have high predictive value. Please note that F1 scores are not a linear measure of performance; for example, a near perfect cluster with precision and recall of 0.99 (almost no erroneous assignments, and almost complete coverage) would get a score of 0.99; a cluster with recall and precision of 0.5 (which misses half the real associations, and half of its assignments are incorrect) would get a score of 0.5. Although the score difference is less than two-fold, the performance difference is enormous.
3. To derive an overall map performance measure that weights each cluster equally, the average F1 score across the 12 clusters was calculated.

Furthermore, map depth, and the distribution of predictions into confidence classes (prediction stratification) were also evaluated. Map depth is the total number of proteins that passed the quality filters to be included for organellar predictions. For prediction stratification, marker proteins were removed from the set (since markers are used to build the SVM models, they generally have high SVM scores). Non-maker proteins were then sorted into the four prediction confidence classes (see above). The absolute number as well as the proportion of predictions in each class were scored (e.g. Figure 2I, J). Furthermore, overall marker prediction accuracy was

calculated within each confidence class; this varies between maps and methods (e.g. Figure 2H), and may suggest which predictions to include in a high confidence set. As a rule of thumb, predictions in the first two confidence classes are of high quality; hence, the greater the proportion of data in these classes, the better.

Concordance analysis

Map concordance (‘agreement’) was defined as the proportion of identical organellar assignments made by two independent maps. The joint outputs from three replicate maps (the ‘combination data sets’) provided by the different labelling methods were analysed. In all cases, predictions were compared to the output from SILAC deep maps (the most accurate in the set). Importantly, marker proteins were removed prior to the analysis; since they are very likely to have similar predictions across maps, their inclusion could otherwise skew the results.

First, the overlapping set of proteins between the SILAC Deep map and the compared map was determined. Second, the fraction of proteins with identical predictions in both maps, divided by the total number of proteins in the common set, was calculated. This provided the baseline concordance, using 100% of the overlapping data. Third, a quality filter on the predictions was introduced. Each organellar assignment is made with a confidence score (the higher, the better). Predictions made with low confidence scores are also less likely to be concordant between maps. For each protein, the lower of the two prediction scores was determined; map concordance was then calculated as a function of this minimum prediction score. By raising the cut-off, a growing proportion of data is excluded from the comparison, but concordance increases.

A corollary of the concordance analysis was that for all map types, regardless of depth and labelling strategy, predictions with scores >0.5 were highly concordant (typically >95-98%), and with scores >1 extremely concordant (typically >98-99.5%). These score cut-offs also coincide with the first two prediction confidence classes defined above.

Detection of dynamic changes between organellar maps

The detection of protein translocations mostly follows the procedure established in Itzhak et al., 2016, which is briefly recapitulated here. Adaptations for the LFQ and TMT workflows are detailed, as well as several modifications to improve the overall sensitivity and robustness of the test.

Datasets

The dynamic experiment consisted of three control samples, and three cognate samples from HeLa cells treated continuously with EGF for 20 min (Itzhak et al., 2016). The six maps obtained with each labelling strategy were analysed as sets.

Calculation of difference profiles

For each map pair, the normalized profile of the treated map was subtracted from the profile of the cognate control map. Three (5 data point) difference profiles were thus obtained for each protein.

Detection of proteins that move significantly between maps (MR plot analysis)

Differences between maps have two sources: genuine translocations of proteins, and experimental noise. Since most proteins do not move between conditions, the majority of difference profiles approximately follows a multivariate normal distribution. Genuine translocations are detected as multivariate outliers from this distribution. A standard statistical measure to define such outliers is the Mahalanobis distance. Since the latter’s calculation is influenced by the outliers themselves, we used a robust calculation of the distance (the minimum covariance determinant, MCD). Distances follow a Chi-square distribution, which allows the conversion into p-values of likelihood for observing the measured distance by chance. The MCD outlier test is implemented in Perseus software (Tyanova et al., 2016b). A critical parameter for the MCD calculation is the proportion of data to use; whereas previously we have used 0.9, we now recommend 0.75, to increase sensitivity and robustness. Furthermore, the MCD derived p-values can be influenced by which proteins are chosen for the first-round calculations of this iterative process; hence, we recommend to calculate median p-values from at least 11

repeats, to ensure robust scores. A future version of Perseus will allow users to specify the number of iterations; we routinely run 101.

For each protein, the differences from three map pairs provide three p-values for movement. A genuine translocation should have a low p-value in each replicate experiment. To combine the replicates, we recommend choosing the highest of the three p-values. This represents the map pair with the smallest observed change. Since all three map pairs are independent experiments, this p-value is now cubed. The new combined p-value is then adjusted for multiple hypothesis correction using the Benjamini-Hochberg method (rank all p values from lowest to highest, multiply each value by the total number of proteins in the set, and divide it by its rank). This final Q-value is then -log(10) transformed, to obtain M (movement) scores. This modified procedure substantially enhances the sensitivity of outlier detection relative to the previous implementation; in addition, M scores now have directly interpretable meaning (e.g. an M score of 2 is equivalent to a Q-score of 0.01, i.e. a 1:100 FDR for movement detection of proteins with M scores of 2 or more). In the absence of proper FDR control datasets (see below), we recommend the following M scores cut offs: 2 (lenient), 3 (stringent), 4 (very stringent).

Genuine translocations have consistent directions across replicates, and hence similar map difference profiles. As a measure of translocation reproducibility, the Pearson correlation of all pairs of difference profiles (Rep1 vs Rep2, Rep1 vs Rep3, Rep2 vs Rep3) is calculated. The lowest correlation is then chosen as the R (reproducibility) score. Only translocations with highly (positively) correlated profile changes are of interest. In the absence of proper FDR control datasets, we recommend the following R-score cut-offs: 0.68 (lenient), 0.81 (stringent), 0.93 (very stringent).

Please note that R scores are orthogonal to M-scores. Their combination ('MR' plot analysis as in Figure 3) results in very strict filtering, even with 'lenient' M and R score cut-offs.

False discovery rate (FDR) control of MR plot analyses

As described previously (Itzhak et al., 2016), the best way to determine significance cut-offs for a MR plot analysis is to perform a mock experiment (three control maps vs three cognate control maps). No genuine translocations are expected under these conditions. The MR plot analysis is performed as described above. Cut-offs for M and R scores are then simultaneously applied to both mock and treatment datasets, and the number of significant movers in the mock set divided by the number found in the treatment experiment corresponds to the estimated FDR at a given MR score cut off. Cut-offs may then be selected to achieve a desired FDR (e.g. 10%, or 1%); alternatively, the FDR at a desired MR cut-off combination may be calculated.

In the present study, the SILAC and LFQ (fast and deep) analyses were fully FDR controlled. The mock dataset for SILAC was provided by the six SILAC (deep) control maps reported in Itzhak et al. (2016). For LFQ, these SILAC maps were reprocessed using the MaxLFQ algorithm (ignoring the SILAC heavy peptides). In all cases, the number of significant hits in the mock set was scaled by the number of profiled proteins in mock and treatment sets. For TMT data, no FDR control dataset was available. As a proxy, we used the same MR cut-offs chosen for the LFQ and SILAC sets.

Profile scatter analysis

Profile scatter within the 20S core proteasome (14 subunits, PSMA1-7, PSMB1-7) was analysed. Only the profiles from the 'deep' combination datasets (with three biological replicates) were included. For each method, the 3 x 14 (0-1 normalised) profiles were extracted, and the 'average' profiles of each replicate determined. Summed absolute deviations from these averages (Manhattan Distances) were then calculated for each subunit, within each replicate. The scatter of the 42 differences was then plotted.

Principal component analysis

For graphical map representation, weighted normalised profiles of the 941 marker proteins common to the SILAC, LFQ and TMT combination datasets (3 deep maps each) were scaled to unit variance, and jointly subjected to principal component analysis (PCA). Figure 4 shows the projections along PCs 1 and 2 (scores plot). For the LFQ6 dataset, a separate PCA had to be performed, since it had one extra fraction per map. To allow optimum visual comparison, the same region of the LFQ6 scores plot is shown as for the other maps, causing clipping of 48 large protein complex markers outside this region.

Software for statistical analysis and graphics

Statistical analyses, data transformation and filtering were performed in Perseus (Tyanova et al., 2016b), Prism 6 (GraphPad Software), and Microsoft Excel (enhanced with the Real Statistics Resource Pack, http://www.real-statistics.com). Principal component analysis was performed in SIMCA 14 (Umetrics/MKS).

Webpage www.MapOfTheCell.org

We have improved the web interface for our database of human subcellular localization predictions.

Mouse neuron organellar anatomy analysis

Copy number determination

Copy numbers per cell, protein concentrations and cell volumes were estimated with the proteomic ruler approach (Wisniewski et al., 2014), implemented in Perseus software (Tyanova et al., 2016b). Briefly, mass spectrometric protein intensities are scaled to the summed histone intensities. Since the histone to DNA ratio is relatively constant, and the amount of DNA per cell is known, the total histone signal can be converted into an absolute protein quantity per cell. All other protein intensities can be scaled accordingly. Normalization to protein molecular weight then yields copy numbers per cell. Based on an estimated total cellular protein concentration of 200 mg/ml, the method also allows an approximate estimation of cell volume, and hence of protein concentrations (Wisniewski et al., 2014).

The proteomic ruler approach requires determination of a complete proteome as input. For the mouse neurons, we derived this in two different ways. First, we used the conventional approach, and directly processed a sample of whole cell lysates for mass spectrometric analysis ('fast' MS protocol, one sample per proteome). This resulted in a depth of ca. 6,000 quantified proteins (from biological duplicates). As an alternative, we combined the mass spectrometric analyses from all seven fractions from organellar map experiments (Figure 5A); the sum of these fractions corresponds to the total cell content. Mass spectrometric intensities from each fraction were normalized to the sum total, and weighted by relative fraction yields as determined by BCA protein assay. Weighted intensities were then added to derive total intensities for each protein. This resulted in a depth of ca. 9,000 quantified proteins (from biological quadruplicates, preps 2-5). The proteomic ruler was then applied to both datasets ('standard' proteome and 'compound' proteome). Importantly, copy number estimates of proteins identified with both methods were highly consistent (correlation >0.95, slope near 1; Figure S3A). Furthermore, both datasets showed the same expected log-normal distribution, with an extended lower range for the compound full proteome (Figure S3B, C). Hence, it is valid to use the compound proteome for copy numbers. The advantage is that the compound proteome is derived from samples that are already analysed as part of the organellar mapping process; no extra samples are required. The depth is also increased, owing to the cell fractionation. We hence recommend this approach for applications where starting material and/or mass spectrometric measuring time are limited.

For the proteomic ruler analysis, we assumed a ploidy of two for mouse neurons, and normalized intensities by protein molecular weight. All proteomes were normalized separately. Default settings were used for all other parameters. Copy numbers and concentrations were calculated as median values from replicates (in log space).

Global protein distribution analysis

To determine the global distribution of proteins ('low resolution' spatial information), we quantified their abundance in the nuclear, cytosolic and membrane fractions (Figure 5A), similar to our previous analysis in HeLa cells (see Itzhak et al., 2016, for details). Briefly, intensities in each fraction were normalized to the sum total, and weighted by their relative yields (as determined by BCA protein assay). Intensities from membrane fractions (2-6 in Figure 5A) were then combined (total membrane fraction). Intensities in the nuclear, cytosolic or membrane fractions were then summed, and expressed as fractions of 1. Proteins were then classified based on their distribution using arbitrary cut-offs:

Classifier	Distribution
Mostly nuclear	nuclear pool >0.9
Mostly membrane associated	membrane pool >0.8
Mostly cytosolic	cytosolic pool >0.9
Nuclear and Cytosolic	(nuclear + cytosolic pools) >0.95
Membrane associated with cytosolic pool	(membrane +cytosolic pools) >0.95
Unclassified	all other proteins

Please note that some proteins had a global as well as an organellar classifier; in these cases, the organellar classifier describes where the membrane associated pool of the protein is located, and the global classifier reports the cytosolic and/or nuclear proportion of the protein.

Mouse neuron organellar composition analysis

Subcellular localization predictions and copy number information were combined to derive organellar compositions, as described (Itzhak et al., 2016). For each organelle, a list of predicted constituents (Table S4) was prepared. For each protein, the copy number/cell was multiplied with the protein's molecular weight, to obtain the total protein mass/cell. Next, this mass was multiplied by the non-cytosolic fraction of the protein. All weighted masses were summed, to obtain the total protein mass of the organelle (per cell). Each protein's contribution to the organelle's total mass was then expressed as a percentage. Dividing the whole organellar protein mass by the whole cell protein mass yielded the relative contribution of the organelle to the cell.

Since lysis of cells invariably causes disruption of ER and thus exaggerated apparent cytosolic pools of ER lumenal proteins (Itzhak et al., 2016), the non-cytosolic correction factor was set to 1 for ER; this was also done for lysosomal proteins, which are mostly lumenal or integral to the membrane.

Comparative analysis mouse neurons vs HeLa

For a quantitative comparison of cell anatomy, we compared the mouse neuron data of this study with our previously prepared HeLa data (Itzhak et al., 2016). We matched identified proteins by UniProt gene name (which is identical between human and mouse in most cases), and assumed that such matched proteins represent orthologues. We also removed duplicated entries for the same gene name within one organism (eg splice variants of the same protein), keeping only the most abundant entry. After filtering, our proteomes contained 8601 and 8469 proteins for mouse neurons and HeLa, respectively. The complete proteome of both cell types is estimated at >10,000 proteins. However, as our plateauing cumulative abundance analysis shows (Figure 6B), the 'missing' proteins are likely to contribute minimally to overall cell protein mass, and hence will not substantially affect the quantitative composition analysis.

Comparisons were performed at the full proteome and organellar levels. 6,708 proteins were identified in neurons and HeLa; hence 78% (6,708/8,601) of the neuron proteome is shared with HeLa cells. For calculating the protein mass overlap, the respective contributions of each shared protein to total proteins mass were compared; the lower value was chose as the overlap. The summed overlap yielded the overall shared mass. To assess organellar composition overlap, the top ten most abundant proteins from each neuron organelle were matched to their HeLa cell orthologues. For each protein, the respective contributions to total neuron or HeLa compartment protein mass were then compared.

Supplemental References

Akimov, V., Rigbolt, K.T., Nielsen, M.M., and Blagoev, B. (2011). Characterization of ubiquitination dependent dynamics in growth factor receptor signaling by quantitative proteomics. Mol Biosyst *7*, 3223-3233.

Arimoto, K., Burkart, C., Yan, M., Ran, D., Weng, S., and Zhang, D.E. (2014). Plakophilin-2 promotes tumor development by enhancing ligand-dependent and -independent epidermal growth factor receptor dimerization and activation. Mol Cell Biol *34*, 3843-3854.

Bryk, A.H., and Wisniewski, J.R. (2017). Quantitative Analysis of Human Red Blood Cell Proteome. J Proteome Res 16, 2752-2761.

Eskova, A., Knapp, B., Matelska, D., Reusing, S., Arjonen, A., Lisauskas, T., Pepperkok, R., Russell, R., Eils, R., Ivaska, J., *et al.* (2014). An RNAi screen identifies KIF15 as a novel regulator of the endocytic trafficking of integrin. J Cell Sci *127*, 2433-2447.

Fan, Q.W., Cheng, C., Knight, Z.A., Haas-Kogan, D., Stokoe, D., James, C.D., McCormick, F., Shokat, K.M., and Weiss, W.A. (2009). EGFR signals to mTOR through PKC and independently of Akt in glioma. Sci Signal *2*, ra4.

Fang, Z., Takizawa, N., Wilson, K.A., Smith, T.C., Delprato, A., Davidson, M.W., Lambright, D.G., and Luna, E.J. (2010). The membrane-associated protein, supervillin, accelerates F-actin-dependent rapid integrin recycling and cell motility. Traffic *11*, 782-799.

Fogelgren, B., Zuo, X., Buonato, J.M., Vasilyev, A., Baek, J.I., Choi, S.Y., Chacon-Heszele, M.F., Palmyre, A., Polgar, N., Drummond, I., *et al.* (2014). Exocyst Sec10 protects renal tubule cells from injury by EGFR/MAPK activation and effects on endocytosis. Am J Physiol Renal Physiol *307*, F1334-1341.

Fukata, Y., Oshiro, N., Kinoshita, N., Kawano, Y., Matsuoka, Y., Bennett, V., Matsuura, Y., and Kaibuchi, K. (1999). Phosphorylation of adducin by Rho-kinase plays a crucial role in cell motility. J Cell Biol *145*, 347-361.

Galovic, M., Xu, D., Areces, L.B., van der Kammen, R., and Innocenti, M. (2011). Interplay between N-WASP and CK2 optimizes clathrin-mediated endocytosis of EGFR. J Cell Sci *124*, 2001-2012.

He, W., Rose, D.W., Olefsky, J.M., and Gustafson, T.A. (1998). Grb10 interacts differentially with the insulin receptor, insulin-like growth factor I receptor, and epidermal growth factor receptor via the Grb10 Src homology 2 (SH2) domain and a second novel domain located between the pleckstrin homology and SH2 domains. J Biol Chem *273*, 6860-6867.

Holland, S., Coste, O., Zhang, D.D., Pierre, S.C., Geisslinger, G., and Scholich, K. (2011). The ubiquitin ligase MYCBP2 regulates transient receptor potential vanilloid receptor 1 (TRPV1) internalization through inhibition of p38 MAPK signaling. J Biol Chem *286*, 3671-3680.

Hosseinibarkooie, S., Peters, M., Torres-Benito, L., Rastetter, R.H., Hupperich, K., Hoffmann, A., Mendoza-Ferreira, N., Kaczmarek, A., Janzen, E., Milbradt, J., *et al.* (2016). The Power of Human Protective Modifiers: PLS3 and CORO1C Unravel Impaired Endocytosis in Spinal Muscular Atrophy and Rescue SMA Phenotype. Am J Hum Genet *99*, 647-665.

Hu, J., Troglio, F., Mukhopadhyay, A., Everingham, S., Kwok, E., Scita, G., and Craig, A.W. (2009). F-BAR-containing adaptor CIP4 localizes to early endosomes and regulates Epidermal Growth Factor Receptor trafficking and downregulation. Cell Signal *21*, 1686-1697.

Julian, L., and Olson, M.F. (2014). Rho-associated coiled-coil containing kinases (ROCK): structure, regulation, and functions. Small GTPases *5*, e29846.

Kamano, Y., Saeki, M., Egusa, H., Kakihara, Y., Houry, W.A., Yatani, H., and Kamisaki, Y. (2013). PIH1D1 interacts with mTOR complex 1 and enhances ribosome RNA transcription. FEBS Lett *587*, 3303-3308.

Karunagaran, D., Tzahar, E., Beerli, R.R., Chen, X., Graus-Porta, D., Ratzkin, B.J., Seger, R., Hynes, N.E., and Yarden, Y. (1996). ErbB-2 is a common auxiliary subunit of NDF and EGF receptors: implications for breast cancer. EMBO J *15*, 254-264.

Klessner, J.L., Desai, B.V., Amargo, E.V., Getsios, S., and Green, K.J. (2009). EGFR and ADAMs cooperate to regulate shedding and endocytic trafficking of the desmosomal cadherin desmoglein 2. Mol Biol Cell *20*, 328-337.

Kozik, P., Hodson, N.A., Sahlender, D.A., Simecek, N., Soromani, C., Wu, J., Collinson, L.M., and Robinson, M.S. (2013). A human genome-wide screen for regulators of clathrin-coated vesicle formation reveals an unexpected role for the V-ATPase. Nat Cell Biol *15*, 50-60.

Larsson, A.H., Lehn, S., Wangefjord, S., Karnevi, E., Kuteeva, E., Sundstrom, M., Nodin, B., Uhlen, M., Eberhard, J., Birgisson, H., *et al.* (2016). Significant association and synergistic adverse prognostic effect of podocalyxin-like protein and epidermal growth factor receptor expression in colorectal cancer. J Transl Med *14*, 128.

Lewis-Saravalli, S., Campbell, S., and Claing, A. (2013). ARF1 controls Rac1 signaling to regulate migration of MDA-MB-231 invasive breast cancer cells. Cell Signal *25*, 1813-1819.

Liu, K., Jiang, T., Ouyang, Y., Shi, Y., Zang, Y., Li, N., Lu, S., and Chen, D. (2015). Nuclear EGFR impairs ASPP2-p53 complex-induced apoptosis by inducing SOS1 expression in hepatocellular carcinoma. Oncotarget *6*, 16507-16516.

Lorch, J.H., Klessner, J., Park, J.K., Getsios, S., Wu, Y.L., Stack, M.S., and Green, K.J. (2004). Epidermal growth factor receptor inhibition promotes desmosome assembly and strengthens intercellular adhesion in squamous cell carcinoma cells. J Biol Chem *279*, 37191-37200.

Lowenstein, E.J., Daly, R.J., Batzer, A.G., Li, W., Margolis, B., Lammers, R., Ullrich, A., Skolnik, E.Y., Bar-Sagi, D., and Schlessinger, J. (1992). The SH2 and SH3 domain-containing protein GRB2 links receptor tyrosine kinases to ras signaling. Cell *70*, 431-442.

Mader, C.C., Oser, M., Magalhaes, M.A., Bravo-Cordero, J.J., Condeelis, J., Koleske, A.J., and Gil-Henn, H. (2011). An EGFR-Src-Arg-cortactin pathway mediates functional maturation of invadopodia and breast cancer cell invasion. Cancer Res *71*, 1730-1741.

Matsuhashi, S., Hamajima, H., Xia, J., Zhang, H., Mizuta, T., Anzai, K., and Ozaki, I. (2014). Control of a tumor suppressor PDCD4: Degradation mechanisms of the protein in hepatocellular carcinoma cells. Cell Signal *26*, 603-610.

Novellasdemunt, L., Tato, I., Navarro-Sabate, A., Ruiz-Meana, M., Mendez-Lucas, A., Perales, J.C., Garcia-Dorado, D., Ventura, F., Bartrons, R., and Rosa, J.L. (2013). Akt-dependent activation of the heart 6-phosphofructo-2-kinase/fructose-2,6-bisphosphatase (PFKFB2) isoenzyme by amino acids. J Biol Chem *288*, 10640-10651.

Pai, R., Szabo, I.L., Giap, A.Q., Kawanaka, H., and Tarnawski, A.S. (2001). Nonsteroidal anti-inflammatory drugs inhibit re-epithelialization of wounded gastric monolayers by interfering with actin, Src, FAK, and tensin signaling. Life Sci *69*, 3055-3071.

Sakaguchi, K., Okabayashi, Y., Kido, Y., Kimura, S., Matsumura, Y., Inushima, K., and Kasuga, M. (1998). Shc phosphotyrosine-binding domain dominantly interacts with epidermal growth factor receptors and mediates Ras activation in intact cells. Mol Endocrinol *12*, 536-543.

Schmandt, R., Liu, S.K., and McGlade, C.J. (1999). Cloning and characterization of mPAL, a novel Shc SH2 domain-binding protein expressed in proliferating cells. Oncogene *18*, 1867-1879.

Sherrill, J.M., and Kyte, J. (1996). Activation of epidermal growth factor receptor by epidermal growth factor. Biochemistry *35*, 5705-5718.

Soubeyran, P., Barac, A., Szymkiewicz, I., and Dikic, I. (2003). Cbl-ArgBP2 complex mediates ubiquitination and degradation of c-Abl. Biochem J *370*, 29-34.

Tong, J., Taylor, P., and Moran, M.F. (2014). Proteomic analysis of the epidermal growth factor receptor (EGFR) interactome and post-translational modifications associated with receptor endocytosis in response to EGF and stress. Mol Cell Proteomics *13*, 1644-1658.

Vincent, S., and Settleman, J. (1997). The PRK2 kinase is a potential effector target of both Rho and Rac GTPases and regulates actin cytoskeletal organization. Mol Cell Biol *17*, 2247-2256.

Wang, Y., Du, D., Fang, L., Yang, G., Zhang, C., Zeng, R., Ullrich, A., Lottspeich, F., and Chen, Z. (2006). Tyrosine phosphorylated Par3 regulates epithelial tight junction assembly promoted by EGFR signaling. EMBO J *25*, 5058-5070.

Watanuki, Z., Kosai, H., Osanai, N., Ogama, N., Mochizuki, M., Tamai, K., Yamaguchi, K., Satoh, K., Fukuhara, T., Maemondo, M., *et al.* (2014). Synergistic cytotoxicity of afatinib and cetuximab against EGFR T790M involves Rab11-dependent EGFR recycling. Biochem Biophys Res Commun *455*, 269-276.

Wheeler, M., and Domin, J. (2001). Recruitment of the class II phosphoinositide 3-kinase C2beta to the epidermal growth factor receptor: role of Grb2. Mol Cell Biol *21*, 6660-6667.

Xu, C.L., Wang, J.Z., Xia, X.P., Pan, C.W., Shao, X.X., Xia, S.L., Yang, S.X., and Zheng, B. (2016). Rab11-FIP2 promotes colorectal cancer migration and invasion by regulating PI3K/AKT/MMP7 signaling pathway. Biochem Biophys Res Commun *470*, 397-404.

Zhang, X., Belkina, N., Jacob, H.K., Maity, T., Biswas, R., Venugopalan, A., Shaw, P.G., Kim, M.S., Chaerkady, R., Pandey, A., *et al.* (2015). Identifying novel targets of oncogenic EGF receptor signaling in lung cancer through global phosphoproteomics. Proteomics *15*, 340-355.

Zhong, S., Yin, H., Liao, Y., Yao, F., Li, Q., Zhang, J., Jiao, H., Zhao, Y., Xu, D., Liu, S., *et al.* (2015). Lung Tumor Suppressor GPRC5A Binds EGFR and Restrains Its Effector Signaling. Cancer Res *75*, 1801-1814.
